# Predicting *in vitro* single-neuron firing rates upon pharmacological perturbation using Graph Neural Networks

**DOI:** 10.3389/fninf.2022.1032538

**Published:** 2023-01-11

**Authors:** Taehoon Kim, Dexiong Chen, Philipp Hornauer, Vishalini Emmenegger, Julian Bartram, Silvia Ronchi, Andreas Hierlemann, Manuel Schröter, Damian Roqueiro

**Affiliations:** ^1^Bioengineering Laboratory, Department of Biosystems Science and Engineering, ETH Zurich, Basel, Switzerland; ^2^Machine Learning and Computational Biology Laboratory, Department of Biosystems Science and Engineering, ETH Zurich, Basel, Switzerland; ^3^SIB Swiss Institute of Bioinformatics, Zurich, Switzerland

**Keywords:** Graph Neural Network, *in vitro* neural network, pharmacological perturbation, extracellular electrophysiology, single neuron activity, machine learning

## Abstract

Modern Graph Neural Networks (GNNs) provide opportunities to study the determinants underlying the complex activity patterns of biological neuronal networks. In this study, we applied GNNs to a large-scale electrophysiological dataset of rodent primary neuronal networks obtained by means of high-density microelectrode arrays (HD-MEAs). HD-MEAs allow for long-term recording of extracellular spiking activity of individual neurons and networks and enable the extraction of physiologically relevant features at the single-neuron and population level. We employed established GNNs to generate a combined representation of single-neuron and connectivity features obtained from HD-MEA data, with the ultimate goal of predicting changes in single-neuron firing rate induced by a pharmacological perturbation. The aim of the main prediction task was to assess whether single-neuron and functional connectivity features, inferred under baseline conditions, were informative for predicting changes in neuronal activity in response to a perturbation with Bicuculline, a GABA_*A*_ receptor antagonist. Our results suggest that the joint representation of node features and functional connectivity, extracted from a baseline recording, was informative for predicting firing rate changes of individual neurons after the perturbation. Specifically, our implementation of a GNN model with inductive learning capability (GraphSAGE) outperformed other prediction models that relied only on single-neuron features. We tested the generalizability of the results on two additional datasets of HD-MEA recordings–a second dataset with cultures perturbed with Bicuculline and a dataset perturbed with the GABA_*A*_ receptor antagonist Gabazine. GraphSAGE models showed improved prediction accuracy over other prediction models. Our results demonstrate the added value of taking into account the functional connectivity between neurons and the potential of GNNs to study complex interactions between neurons.

## 1. Introduction

Graph Neural Networks (GNNs) constitute a type of neural networks that feature node or graph representations of relational information in the respective graph structures (Scarselli et al., 2009). The ability of GNNs to model structural or relational information has led to successful applications over a wide range of topics (Zhou et al., 2020) including physics (Battaglia et al., 2016; Sukhbaatar et al., 2016; Hoshen, 2017; Watters et al., 2017; Kipf et al., 2018; Sanchez-Gonzalez et al., 2018), biology (Fout et al., 2017; Rhee et al., 2018; Zitnik et al., 2018), chemistry (Cortes et al., 2015; Kearnes et al., 2016; Do et al., 2019), and other application areas, such as traffic forecasts (Yu et al., 2018; Guo et al., 2019; Cui et al., 2020; Zheng et al., 2020), recommendation systems (van den Berg et al., 2017; Ying et al., 2018; Fan et al., 2019; Wu et al., 2019), and stock market prediction (Matsunaga et al., 2019; Yang et al., 2019). In neuroscience, GNNs have recently been shown to be effective in several tasks, such as classification of brain states (Bessadok et al., 2019; Banka et al., 2020; Lostar and Rekik, 2020; Cui et al., 2021; Li et al., 2021; Wein et al., 2021; Xing et al., 2021), detection of the default mode network (Wang et al., 2022), brain parcellation (Eschenburg et al., 2021; Qiu et al., 2022), and disease detection (Chen et al., 2021; Chan et al., 2022) based on functional connectivity derived from functional magnetic resonance imaging data. At the neuron level, GNNs were used to model motor action trajectories in *C. elegans* using connectivity graphs derived from calcium imaging of individual neurons (Wang et al., 2021).

In this study, we seek to leverage well-established GNN models to predict single-neuron firing rate responses to pharmacological perturbation using features extracted from extracellular electrical activity of neurons in the baseline state. We therefore obtained spike train/waveform-derived single-neuron features and functional connectivity inferred from large-scale recordings of primary rodent neuronal cultures by means of high-density microelectrode arrays (HD-MEAs). To perturb neuronal networks, we applied Bicuculline (BIC; GABA_*A*_ antagonist, 5μM), a widely used pharmacological compound to study induced excitation in neural circuits (Eisenman et al., 2015; Ciba et al., 2020). We hypothesized that combining single-neuron features and functional connectivity using GNNs would improve prediction of single-neuron firing rate changes observed during the perturbation over prediction made using only single-neuron features.

Our hypothesis is based on the well-documented involvement of excitatory and inhibitory neurons and their connectivity in modulating ongoing neuronal network dynamics (Buzsáki, 2010; Landau et al., 2016), and the notion that extracellular action potential (AP) waveform features may be used to differentiate between different types of neurons. Specifically, previous works have demonstrated that AP waveform features combined with single-neuron firing patterns can be used to classify functionally distinct neurons recorded *in vivo* (i.e., differences in the width of AP waveforms and the shape of spike train auto-correlograms; Mosher et al., 2020; Petersen et al., 2021). However, the degree to which AP waveforms of *in vitro* grown neurons are indicative for excitatory/inhibitory cell-types is the subject of an ongoing debate (Weir et al., 2014). Among different types of connectivity, functional connectivity (FC), here broadly defined as the statistical co-activity between neurons (Stephan and Friston, 2009; Feldt et al., 2011), has been excessively used to study characteristics of neuronal circuits (Friston, 1994; Greicius et al., 2003; Damoiseaux et al., 2006; Cohen and Maunsell, 2009; Shirer et al., 2012; English et al., 2017; Pastore et al., 2018). In this study, we considered two undirected measures and one directed measure that describe pairwise co-activity between neurons: the Pearson Correlation Coefficient (PCC), the Spike Time Tiling Coefficient (STTC; Cutts and Eglen, 2014) and Cross-Correlation Histograms (CCHs; Eggermont, 2010).

Our analysis revealed that GNN models showed improved prediction accuracy while models that did not utilize connectivity information yielded a prediction accuracy close to that of the baseline model, which used the mean of the target variables in the training dataset to predict target variables of the testing dataset. These findings demonstrate the advantage of studying network activity by using a combined model of single-neuron features and neuron-to-neuron connectivity information.

The paper is organized as follows: First, we introduce the HD-MEA experiments and provide results on the observed single-neuron electrophysiological features and characterize spontaneous population activity. Next, we quantify the firing-rate changes of neurons during the BIC (5μM) perturbation and compare the performance of different machine learning models in predicting single-neuron firing rate changes following BIC application. Finally, we test the generalizability of the results by extending the analysis to two additional datasets - one dataset including cultures perturbed with BIC and another dataset with cultures perturbed with the compound Gabazine (GBZ), another GABA_*A*_ antagonist (5μM).

## 2. Materials and methods

### 2.1. Cell culture and plating

Primary rat neurons were obtained from the dissociated hippocampus of Wistar rats at embryonic day (E) 18, using the protocol previously described in Ronchi et al. (2019). All animal experimental protocols were approved by the Basel-Stadt veterinary office according to Swiss federal laws on animal welfare and were carried out in accordance with the approved guidelines. Prior to cell plating, HD-MEA chips were sterilized in 70% ethanol for 30 min. Then, ethanol was removed and the chips were rinsed three times with distilled sterile water and left to dry. The HD-MEA chips were then coated with a layer of 0.05% polyethylenimine (Sigma-Aldrich, Buchs, Switzerland) in a borate buffer (Thermo Fisher Scientific, Waltham, MA, United States) to render the surface more hydrophilic. Prior to cell plating, a thin layer of laminin (Sigma-Aldrich, 0.02 mg/mL) in Neurobasal medium (Gibco, Thermo Fisher Scientific) was pipetted onto the array and incubated for 30 min at 37°C to promote cell adhesion. We dissociated hippocampi of E18 Wistar rat enzymatically in trypsin with 0.25% EDTA (Gibco), followed by trituration. Cell suspensions of 12,000−15,000 cells in 7 μL were then plated on top of the electrode arrays. The plated chips were incubated at 37°C for 30 min before adding 2 mL of the plating medium. The plating medium consisted of Neurobasal, supplemented with 10% horse serum (HyClone, Thermo Fisher Scientific), 0.5 mM Glutamax (Invitrogen, Thermo Fisher Scientific), and 2% B-27 (Invitrogen). After 5 days, 50% of the plating medium was replaced with a growth medium, containing Brainphys medium supplemented with SM1 and N2-A (Stemcell technologies, Cologne, Germany). For the rest of the experiments medium changes were performed twice a week using the same Brainphys-based medium. The chips were kept inside a humidified incubator at 37°C and 5% CO_2_/95% air.

### 2.2. High-density microelectrode array recordings

In this study, we obtained two different HD-MEA datasets: a dataset to first probe they hypothesis of the main experiment (later referred to as “main dataset”) and a dataset to test the generalizability of the findings of the main experiment (later referred to as the “test dataset”). For the main dataset, electrophysiological recordings were obtained using a published CMOS-based high-density microelectrode array (HD-MEA; Müller et al., 2015). This HD-MEA features 26,400 electrodes arranged in a 120 x 220 electrode grid with a microelectrode center-to-center spacing (pitch) of 17.5 μm; the overall sensing area of this HD-MEA is 3.85 x 2.10 mm^2^. The HD-MEA enables simultaneous recording of up to 1,024 electrodes at a sampling rate of 20 kHz. Recordings were performed inside an incubator at 37°C and 5% CO_2_/95% O_2_ and were made at DIVs 22–25. Each recording started with a whole-array “activity scan” to determine the active electrodes on the HD-MEA. The activity scan consisted of 29 dense electrode configurations to scan through the entire sensing area of the electrode array; each configuration was sequentially recorded for 60 s. From the activity scan, up to 1,024 electrodes were selected by prioritizing electrodes with high firing rates (based on online detected multi-unit activity). Next, *k*-means clustering (*k* = 4) was applied to the coordinates of the chosen electrodes to get four centroids (center position of each cluster) - and based on these centroids - four non-overlapping, rectangular dense configurations with 2 × 2 sparsity (center-to-center spacing of 35 μm, two electrodes apart) were created. The computed centroids were checked manually again before creating the dense configurations (later referred to as “sub-networks”) and shifted, if necessary, to capture most of the selected electrodes (Supplementary Figures 1, 2). For each configuration, nine longitudinal recordings were acquired. The first two recordings were combined and used as a baseline, and the following seven recordings were used to measure the perturbation response. The duration of each sub-network recording was 20 min so that a full run of recordings through four dense configurations lasted 80 min. The interval between each run of recording was 2 h. For the test dataset, we performed recordings with commercially available 6-well HD-MEA plates (MaxTwo system by Maxwell Biosystems, Zurich, Switzerland). Each MaxTwo well features an HD-MEA, as described for the main dataset, however the sampling rate of these recordings was lower (10 kHz). For this dataset electrodes featuring higher firing rates were prioritized from the activity scan (up to 1,020 of the most active recording electrodes were selected as the network configuration). Again, we recorded a baseline condition (2 h) and obtained two recordings for the perturbation conditions (20 min per recording, 2 h spacing in between).

### 2.3. Pharmacology

For the main dataset/experiment, the GABA_*A*_ antagonist Bicuculline (BIC; Abcam, Cambridge, United Kingdom) was pipetted directly from the stock solution into the culture medium to generate a 5μM solution. The concentration was selected based on the result reported in Ueno et al. (1997). Recordings started 5 min after the application of the drug. For the test dataset/experiment, we recorded the perturbation response of neuronal cultures following BIC, and following Gabazine (GBZ; Abcam, Cambridge, United Kingdom). GBZ (5μM) was introduced following the same procedure stated in the description of the main dataset.

### 2.4. Data preprocessing

#### 2.4.1. Spike-sorting and quality control of sorted units

For each HD-MEA/well, all available recording time points for the same configuration were concatenated, filtered, and spike-sorted using “Kilosort2” (Pachitariu et al., 2016); the applied parameters are stated in Supplementary Table 1. To be included in subsequent analyses, all inferred spike-sorted units had to pass a quality control: First, we removed units with a firing rate below 0.05 Hz and higher than 30 Hz (measured across both the baseline and the full recording). The lower bound for the firing rate was necessary to ensure reliable feature extraction (see Section 2.4.2.2). Then we computed the refractory period violation ratio, which was calculated as the fraction of interspike intervals (ISIs) <2 ms (Hill et al., 2011). We therefore inferred the number of spikes within the [±2 ms] bins of the spike train autocorrelogram (ACG) and then computed the fraction between this count and the total number of spikes in a larger range of the ACG [±50 ms]. Any template exceeding a refractory period violation ratio of 0.3 were removed. Next, we extracted action potential waveform features and firing pattern features for each unit (see Section 2.4.2.1). The extracted features were later also used to apply further filtering operations and to select units with a peak-waveform resembling a somatic or axon initial segment origin (Bakkum et al., 2019). Based on these preprocessing steps, the obtained units were considered to originate from single neurons.

#### 2.4.2. Single-neuron extracellular feature extraction

**Figure 1:**
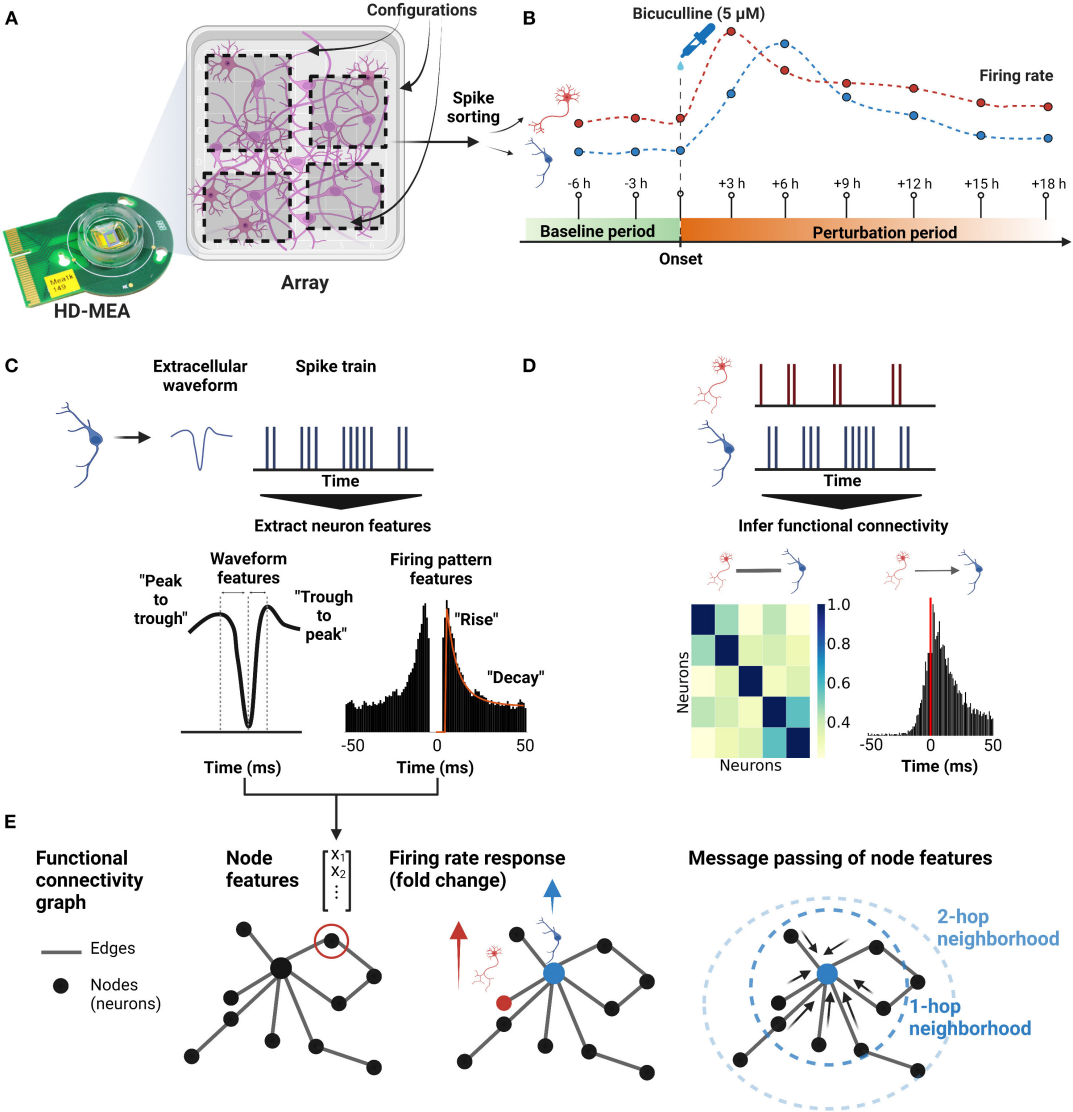
Overview of the experiment. **(A)** Dissociated primary neurons from rat hippocampi were cultured on HD-MEAs until the neurons reached a mature state, i.e., days *in vitro* (DIV) 21. For each HD-MEA, four dense electrode configurations were defined to capture most of the network activity. **(B)** Using the same configurations, nine consecutive recordings were acquired with the first two recordings being the baseline recordings and the other seven recordings measuring the perturbation response (BIC, 5μM). For each configuration (sub-network), all nine recordings were concatenated and then spike-sorted to obtain extracellular waveforms and spike trains of the neurons. From the spike train of each neuron, the firing rate trajectory of each neuron throughout the perturbation window was reconstructed. **(C)** To characterize each neuron, four extracellular waveform features and three firing pattern features were extracted from the spike-sorted outputs. **(D)** Both, undirected and directed FCs were inferred between spike trains of neurons. **(E)** Connectivity graphs were constructed based on the FCs, and each neuron in the graph had seven single-neuron features obtained from step **(C)** as node features. GNN models were then used to predict firing rate changes of individual neurons using the node features and the inferred functional connectivity.

The extraction of single-neuron extracellular features followed mainly the “cell explorer” work flow as described in Petersen et al. (2021), and Matlab (version R2019b, Mathworks, Natick, Massachusetts, United States) was used to extract the single-cell features from the spike-sorted units, respectively their so-called “electrical footprint” on the HD-MEA (see Figure 1).

##### 2.4.2.1. Action potential waveform features

For each unit, we sampled 300 spikes and then averaged the extracellular waveforms - comprising the electrical footprint of the unit on the HD-MEA. The averaged waveforms were then filtered using a 3rd order highpass filter with a lower bound of 500 Hz (Petersen et al., 2021). The electrode featuring the filtered waveform with the largest amplitude, the “peak electrode,” was selected for all further analysis. We used the unfiltered averaged waveform from the selected peak electrode to compute waveform features. Before extracting waveform features, we up-sampled the waveforms by a factor of 2 (4 for the test dataset) using spline interpolation, and *z*-transformed the up-sampled waveforms. For example, 81 time points (corresponding to 4 ms at 20 kHz sampling rate) were upsampled to 162 time points. The extracted waveform features included the peak-to-trough duration, the trough-to-peak duration, the AB ratio, and the action potential half width (Supplementary Figure 3). ‘Peak-to-trough” was defined as the time from the peak (local maximum before the minimum of the trough) to the post-hyperpolarization peak (global minimum, trough). “Trough-to-peak” was the time from the minimum of the trough (global minimum) to the post-hyperpolarization peak (local maximum after the trough). “AB ratio”(or “waveform peak to peak ratio”) was defined as the ratio between the amplitude of the peak before the trough (A) and the size of the peak after the trough (B), AB ratio = $\frac{\left(\text{B}-\text{A}\right)}{\left(\text{B}+\text{A}\right)}$. In addition, we inferred the action potential half width, which was calculated as the width of the trough at half the peak amplitude.

Templates that showed very wide trough widths (peak to trough + trough to peak > 1.5 ms) and a high degree of asymmetry (AB ratio < −0.3 or AB ratio > 0.6) were removed from downstream analysis, and the thresholds were selected based on previously reported values (Peyrache et al., 2012; Peyrache and Destexhe, 2019; Petersen et al., 2021).

##### 2.4.2.2. Firing pattern features

Spike times from the baseline recordings (main dataset: two time points, 40 min duration in total; test dataset: 2 h in duration) were used to extract the single-cell firing patterns. These features were computed based on ISI histograms and ACGs of individual units. ISI histograms were computed with a bin size of 1 ms and considered up to 100 bins (100 ms). Similarly, for the ACGs, the bin size was 1 ms, and a time window of 100 ms [±50 ms] was considered. Firing pattern features were the single-cell “burstiness” (Mizuseki and Buzsáki, 2013), τ_rise_ and τ_decay_, where each time constant (τ) was the time constant modeling the rise/decay of the ACG (Supplementary Figure 3). Single-cell burstiness was computed based on the ISI histograms and was defined as the number of spikes occurring within 6 ms bins of ISI histograms divided by the total amount of spikes in the ISI histogram. For the time constants τ_rise_ and τ_decay_, the ACGs were fitted with the following triple exponential function to characterize the firing pattern of a neuron (Petersen et al., 2021).

$$\begin{array}{l} \text{AC}{\text{G}}_{\text{fit}}=\max(c\cdot \exp^{-\frac{x-t_{\text{refrac}}}{\tau_{\text{decay}}}}-d\cdot \exp^{-\frac{x-t_{\text{refrac}}}{\tau_{\text{rise}}}}\\ \;+h\cdot \exp^{-\frac{x-t_{\text{refrac}}}{\tau_{\text{burst}}}}+{\text{rate}}_{\text{asymptote}},0) \end{array}$$

where τ_burst_ and rate_asymptote_ were additional parameters in the exponential function to facilitate the fitting to the ACGs.

To fit the equation, the refractory period (*t*_refrac_) was first computed using the method defined by Royer et al. (2012). For each ACG of a neuron, the instantaneous derivative (computed with the “diff” function in Matlab) from the 0 ms bin to the time bin where the count was at a maximum (peak bin) was computed, and the standard deviation of the derivative values was computed. The refractory period was defined as the first time bin where the derivative exceeded one standard deviation. Then, the ACG was smoothed with the moving-average filter spanning 5 ms using the “smooth” function in Matlab, and the previously calculated refractory period time bins of the smoothed ACG were set to zero. The smoothed ACG was then fitted with the above exponential equation to obtain ACG fits using the “fit” Matlab function. Any neuron that showed a poor fit with an *r*-square value lower than 0.8 was removed from the downstream analysis (Supplementary Figures 4, 5).

#### 2.4.3. Clustering of single-neuron features for visualization

**Figure 2:**
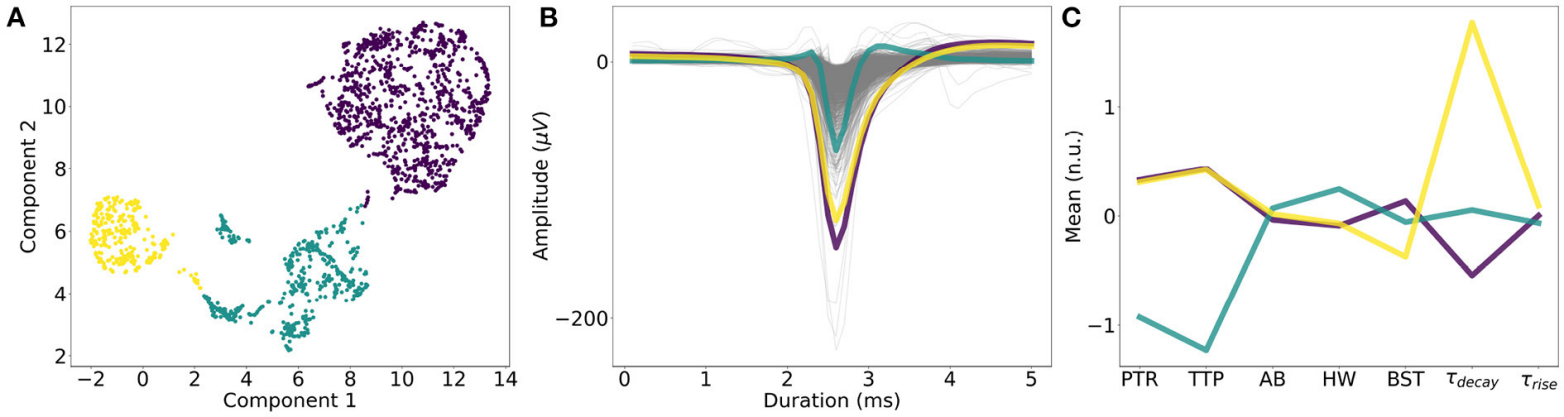
Single-neuron waveform features. **(A)** *k*-means clustering of dimensionality-reduced (UMAP) single-neuron features. *k* = 3 (number of clusters) yielded the best silhouette score. **(B)** Average waveforms and single-neuron features for each cluster. **(C)** One cluster (in cyan) showed smaller average PTR (“Peak-to-trough”) and TTP (“Trough-to-peak”) values, compared to the other two clusters (yellow and purple), while the other two clusters mainly differed in “τ_decay_,” which is the time constant characterizing the downward slope of the ACG. “AB,” “HW,” “BST” denotes “AB ratio,” “Halfwidth,” and “Burstiness.” respectively. “τ_rise_” is a measure of the upward slope of the ACG.

The extracted single-neuron features were scaled, and Uniform Manifold Approximation and Projection (UMAP) dimensionality reduction (McInnes et al., 2018) was performed on the scaled features. The Python library “umap” was used with the default parameters to generate a two-dimensional embedding. Clustering with *k*-means was performed with varying *k* (number of clusters) in the range of [2, 12] in increments of one. For displaying purposes (Figure 2), we selected the *k* with the highest silhouette score (Rousseeuw, 1987; Supplementary Table 2).

### 2.5. Functional connectivity measures

Different functional connectivity measures were inferred from the quality-controlled spike times of the baseline network recordings, i.e., before the application of any perturbation.

#### 2.5.1. Pearson correlation coefficient

To calculate Pearson correlation coefficients between units, the spike times were binned. Assuming the synaptic delay time window to be <20 ms (Izhikevich, 2006), a bin size of 20 ms was used. Pearson correlation between a neuron pair (*i, j*) was then computed as follows:

$$\begin{array}{l} \text{C}\left(i,j\right)=\frac{\langle b_{i}-m_{i},b_{j}-m_{j}\rangle }{\sqrt{\langle b_{i}-m_{i},b_{i}-m_{i}\rangle \cdot \langle b_{j}-m_{j},b_{j}-m_{j}\rangle }}, \end{array}$$

where 〈., .〉 denotes a scalar product between two vectors; *b*_*i*_, *m*_*i*_ are the binned spike train and the average of the binned spike train of neuron *i*, respectively. To correct for spurious correlation values, surrogate spike trains were generated by shuffling spike times while keeping the inter-spike intervals [“shuffle-isis,” “Elephant” Python package (Denker et al., 2018)]. From the correlation value of the experimental data we subtracted the maximum correlation value obtained from 100 instances of randomly shuffled spike trains. If the experimental correlation value was smaller than the maximum of the surrogates, then the value was set to zero. Negative correlations from the experimental data were not considered. As a result, an undirected, weighted functional connectivity matrix was generated with each connection being surrogate-subtracted and positive. Neurons without connections were discarded from the downstream analysis to ensure better training of GNN models.

#### 2.5.2. Spike Time Tiling Coefficient

The Spike Time Tiling Coefficient (STTC) between a neuron pair (*i, j*) was computed as defined in Cutts and Eglen (2014).

$$\begin{array}{l} \text{STTC}\left(i,j\right)=\frac{1}{2}\cdot \left(\frac{P_{ij}-T_{j}}{1-P_{ij}T_{j}}+\frac{P_{ji}-T_{i}}{1-P_{ji}Ti}\right), \end{array}$$

where *P*_*ij*_ was defined as the number of spikes from neuron *i* that lie within [-dt,+dt] of the spikes of neuron *j* divided by the total number of spikes from neuron *i*. *T*_*i*_ is a relevant time window for neuron *i*, defined as the fraction that the summed time window [-dt,+dt] accounts for the entire recording duration. For comparability with the Pearson correlation, the time window length dt was set to 10 ms to match the bin size of 20 ms for Pearson correlations. We used the STTC implementation of the Python “Elephant” package (Denker et al., 2018). To prevent spurious connections, surrogate spike trains were generated to compute surrogate STTC values. As negative STTC values, resulting from the experimental spike trains, could not be distinguished from the values from surrogate trains, negative values were discarded. Among positive values, only positive surrogate-subtracted values were considered as valid STTC values. Therefore, the resulting adjacency matrix was undirected and weighted with strictly positive entries. As done with PCC, neurons without connections were discarded from the downstream analysis.

#### 2.5.3. Directed functional connectivity inference using cross-correlation histograms (CCHs)

Inference of directed connectivity was performed by adapting a previously described method (Pastore et al., 2018). Briefly, pairwise cross-correlation histograms were computed with a bin size of 1 ms for a window duration of 50 ms [±25 ms]. We selected a reasonably small bin size of 1 ms to compute cross-correlation histograms as variability in the bin counts resulting from smaller bin sizes was suggested to negatively impact the inference of inhibitory connections (Pastore et al., 2018). Using counts in the 25-ms postsynaptic window of the cross-correlation histogram, the average bin count was subtracted from each bin to normalize the pairwise activity. The duration of the postsynaptic window was set to capture the fast monosynaptic delay of synaptic transmission (2 − 10 ms; Suresh et al., 2016).

$$\begin{array}{l} {\hat{\text{C}}}_{ij}\left(t\right)={\text{C}}_{ij}\left(t\right)-\frac{1}{B}\overset{B}{\underset{v=1}{\sum }}C_{ij}\left(v\right) \end{array}$$

${\hat{\text{C}}}_{ij}\left(t\right)$ and C_*ij*_(*t*) denote the normalized and non-normalized postsynaptic bins of the cross-correlation histogram. *B* is the number of postsynaptic bins considered for inference (*B* = 25, for 25 ms). The absolute maximum and the time bin of the maximum were noted for each pairwise correlation histogram. If the absolute maximum was in the trough, then the pair was tentatively labeled as “inhibitory,” and if the absolute maximum was at the peak then the pair was labeled as “excitatory.” Assuming an axonal propagation velocity of 400 mm/s, a pair was considered a spurious connection if the distance between a labeled neuron pair was longer than the distance reachable within the peak timing. Adapting the implementation of Pastore et al. (2018), these labeled pairs were then hard-thresholded with peer-based thresholds. For example, for both excitatory and inhibitory pairs, μ+σ (mean + 1 standard deviation of all pairs) was applied as a threshold to binarize connections as connected or non-connected. In the present study, we introduced an additional sensitivity parameter γ, and applied five sets of hard thresholds μ + σγ with γ = 0, 0.5, 1, 1.5, 2.0. This step was necessary to probe directed functional connectivity without knowing the ground-truth connectivity of the experimental data. Given the thresholded adjacency matrix, Dale (1935)'s law was applied by checking the number of excitatory/inhibitory connections each neuron has, and by discarding the minority of putatively mislabeled excitatory or inhibitory connections. Finally, as GNNs are known to excel at modeling connected graphs (Hamilton et al., 2017), we only kept the largest component for the analysis. As a result, a set of two directed, unweighted adjacency matrices were generated representing excitatory and the inhibitory connectivity, respectively for each γ.

### 2.6. Network selection criteria and network activity characterization

#### 2.6.1. Network selection criteria

From the inferred FCs, we computed the number of (dis-)connected components. All networks that contained 30 or more neurons (nodes) were considered for the prediction task and further network-activity characterization.

#### 2.6.2. Graph metrics for functional connectivity graphs

The graph size of a FC graph was defined by the number of neurons (nodes) in the graph. The average physical distances between neurons were computed with respect to the location of the peak electrode, i.e., the electrode featuring the largest signal amplitude within the electrical footprint of the unit. Then, the physical distance between neurons was defined as the Euclidean distance between the respective peak electrodes. The degree strength of a neuron *i* was defined as the column sum of the *i*-th column in FC graphs. The shortest path between neuron *i, j* was defined as the number of edges that was required to reach from neuron *i* to neuron *j* given the respective distance. For weighted FCs, distance matrices were generated by inverting each element (correlation value) in the FC graphs. Subsequently, the average shortest path in a graph was defined as the average over all shortest paths between the neurons in the graph. Degree strength and shortest path were computed using the Python library ‘bctpy.”

#### 2.6.3. Participation ratio

To measure how correlated the firing activity was for each network, we computed the Participation Ratio (PR); it was here used as a normalized variant (PR/number of neurons). The computation and interpretation of the PR was based on an adapted implementation of the method of Recanatesi et al. (2020). First, the spike trains of the baseline recordings were binned (20 ms window size). The binned spike trains were *z*-transformed and used to compute inner products and to generate a correlation matrix. Eigenvalues were collected from the eigendecomposition of the matrix to measure the correlated activity between neurons. The participation ratio was defined as

$$\begin{array}{l} PR=\frac{{\left(\sum_{i}\lambda_{i}\right)}^{2}}{\sum_{i}\lambda_{i}^{2}}, \end{array}$$

where λ_*i*_ is *i*-th eigenvalue of the correlation matrix. The resulting *PR* value indicates the number of principal components that are necessary to explain 80–90% of the total variance for typical Principal component analysis (PCA) eigenspectra (Gao et al., 2017). In this study we normalized the PR by dividing it by the number of neurons (*N*) in the network.

$$\begin{array}{l} \frac{1}{N}\leq \text{PR normalized}\leq 1 \end{array}$$

A normalized *PR* value of <0.8, suggests that the majority of the variance in the network activity could be explained by <80% of principal components (Stigler, 1955; Gao et al., 2017; Recanatesi et al., 2020).

### 2.7. Quantification of firing rate changes

Perturbation-induced changes in both network and single-neuron firing rates were measured in x-fold changes. For the overall network activity, we directly compared the maximum perturbation response of the network firing rate (maximum population firing rate in a given perturbation window) to the baseline network firing rate. Therefore, the x-fold change was defined as $\Delta_{fch}=\frac{\text{maximum FR}}{\text{baseline FR}}$. For single neurons, we measured x-fold changes based on the difference between maximum firing rate and baseline firing rate: $\Delta_{fch^{\prime }}=\frac{\text{maximum FR}-\text{baseline FR}}{\text{baseline FR}}$. In the case of single neurons, the maximum FR was computed in two different time windows during the perturbation period: (1) immediately after perturbation, i.e., in the first two recordings after perturbation, and (2) during the entire perturbation period. These two conditions correspond to the two prediction tasks described in Section 3.3. This modification of the x-fold change was necessary to highlight the representation of neurons that decreased their firing rates during the perturbation. Subsequently, the differential x-fold changes were used as target variables for the prediction tasks. For the test dataset, we only considered the comparison between baseline and immediate perturbation response.

### 2.8. Prediction of single-neuron firing rate responses

#### 2.8.1. Model training and testing

For all following prediction models, input variables and target variables were standard-scaled. For every train-test split, a standard scaling operation was performed on the train split, and the fitted scaler was used on the test split to scale the data. For all datasets, nested leave-one-out cross-validations were performed. The partition of the training/testing data was performed identically for all prediction models. Whenever a specific network was held out for testing, networks from the same HD-MEA chip were also excluded from the training set. This approach poses an inductive (or out-of-distribution) prediction task, which is particularly useful to understand the transferability of the model to new unseen networks. The performance of each prediction model was evaluated based on the average of mean squared errors (MSEs) resulting from all networks in the respective dataset. For the random forest regression model and GNN models, the MSE for each network was computed by taking the mean of 30 runs to account for the inherent stochasticity during the training.

#### 2.8.2. The baseline model and prediction models without functional connectivity information

As a baseline model to compare performance of each model, the average of the target variables from the training split was computed and used as the prediction value to compute the MSE. To find out whether there was a linear correlation between the input features (“peak-to-trough,” “trough-to-peak,” “AB ratio,” “half width,” “burstiness,” “τ_decay_,” and “τ_rise_”) and the target variables ($\Delta_{fch^{\prime }}$), we fitted a linear regression model with the Python package “Scikit-learn” (Pedregosa, 2011), using default parameters. To measure potential non-linear interactions between input features and target variables, a random forest regression model was fitted using “Scikit-learn.” A grid search was performed to select the best model using the sets of parameters stated in the Supplementary Table 3.

#### 2.8.3. Graph convolutional network models including functional connectivity information

In this study, we applied three types of graph convolutional network models: Graph Convolutional Network (GCN; Kipf and Welling, 2016), GraphSAGE (Hamilton et al., 2017), and Relational Graph Convolutional Network (RGCN; Schlichtkrull et al., 2018).

First, we implemented the Graph Convolutional Network (GCN). We denote the undirected, weighted graph as *G* = (*V, E*) with *N* nodes *v*_*i*_ ∈ *V*, edges (*v*_*i*_, *v*_*j*_) ∈ *E*, a weighted adjacency matrix *A* ∈ ℝ^*N* × *N*^ and a degree matrix $D_{ii}=\underset{j}{\sum }A_{ij}$. Then, the convolution operation was defined as

$$\begin{array}{l} X^{\prime }={\hat{D}}^{-1/2}Â{\hat{D}}^{-1/2}X\Theta , \end{array}$$

where *X*′ was the output matrix and *X* was the input matrix with Â = *A* + *I*, ${\hat{D}}_{ii}=\underset{j=0}{\sum }{Â}_{ij}$ and Θ being the trainable parameter matrix. A node-wise computation can be written as

$$\begin{array}{l} {\text{x}}_{i}^{\prime }=\Theta \cdot \underset{j\in N\left(i\right)}{\sum }\frac{e_{ji}}{\sqrt{\hat{d_{j}}\hat{d_{i}}}}\cdot {\text{x}}_{j}, \end{array}$$

where *e*_*ji*_ was the edge weight from source node *j* to target node *i* with ${\hat{d}}_{i}=1+\underset{j\in N\left(i\right)}{\sum }e_{ji}$ and *N*(*i*) was the neighborhood of node *i*.

As a complementary method to GCN, GraphSAGE (Hamilton et al., 2017) was implemented with two types of pooling operations: mean pooling and max pooling. The convolution operation of the GraphSAGE model for each node is given as

$$\begin{array}{l} {\text{x}}_{i}^{\prime }=\Theta_{\text{bias}}\cdot {\text{x}}_{i}+\Theta \cdot \underset{j\in N\left(i\right)}{\text{AGGREGATE(max, mean)}}\left(e_{ji}\cdot {\text{x}}_{j}\right), \end{array}$$

where *e*_*ji*_ was the edge weight from source node *j* to target node *i* and Θ_*bias*_, Θ being trainable matrices for an additive bias and aggregated message respectively. *N*(*i*) denoted the neighborhood of node *i*.

Finally, for directed FC graphs, we modeled two distinct relations (excitatory, inhibitory) adapting the original implementation of a Relational Graph Convolutional Network (RGCN) model (Schlichtkrull et al., 2018). As input to the RGCN we used the directed, labeled multi-graphs as *G* = (*V, E, R*) with nodes *v*_*i*_ ∈ *V* and labeled edges (*v*_*i*_, *r, v*_*j*_) ∈ *E*, where *r* ∈ *R* is a relation type. The node-wise convolution operation of relational information was then given as

$$\begin{array}{l} {\text{x}}_{i}^{\prime }=\Theta_{\text{bias}}\cdot {\text{x}}_{i}+\underset{r\in R}{\sum }\underset{j\in N_{r}\left(i\right)}{\sum }\frac{1}{N_{r}\left(i\right)}\Theta_{r}\cdot {\text{x}}_{j}, \end{array}$$

where both Θ_bias_, Θ_*r*_ were trainable parameter matrices for an additive bias and aggregated tensor based on the relations. *N*_*r*_(*i*) denoted indices of neighbors of node *i* with the relation *r*.

For all three GNN models, the convolution operation was repeated *n* times to aggregate information from *n*-hop neighborhoods. We trained multiple models with up to *n* = 3 convolution layers, corresponding to the 3-hop neighborhood. After each convolution operation, a dropout layer and a Rectified Linear Unit (ReLU) activation layer followed. Node embeddings generated from each layer were concatenated and then passed through a linear layer to predict target variables. All models were implemented using the Python library “pytorch geometric” (Fey and Lenssen, 2019). For all graph convolutional network models, a grid search was performed for the model selection using the parameter sets stated in Supplementary Table 4.

## 3. Results

### 3.1. Overview on experimental procedures and datasets

In this study, we investigated whether the joint representation of extracellular single-neuron electrophysiological features and functional connectivity, inferred from ongoing spontaneous neuronal activity, allows the prediction of changes in firing activity induced by a pharmacological perturbation. We therefore plated neurons derived from dissociated embryonic rat hippocampi onto HD-MEAs, cultured them until DIV 21 (Figure 1A), and performed whole-array activity scans to screen for neuronal activity (see Section 2). Next, we defined four dense electrode configurations per HD-MEA (electrode center-to-center pitch of 17.5 μm, 746 ± 78 electrodes (μ ± σ) per configuration, later referred to as “sub-networks”) and recorded electrical neuronal activity of highly populated areas across a baseline period (2 recordings, 20 min each). As a next step, we perturbed cultures with BIC (5μM), and tracked their responses in activity (seven recording session, approx. 18 hours; Figure 1B). After the experiment, we spike-sorted the HD-MEA data, and following a quality control step (see Section 2), we inferred single-neuron spike trains, extracellular waveform features and functional connectivity (Figures 1C, D).

The main dataset of this study consisted of 24 sub-networks from eight different HD-MEAs, resulting in a total of 1,695 neurons that were pooled across chips and used for subsequent analyses. Next, we constructed and trained different GNN models to predict firing rate changes of neurons using a joint representation of node features and functional connectivity graph (Figure 1E). We focused on functional connectivity measures that were simple to implement and output denser graphs. We also probed the usefulness of a more sophisticated statistical model, namely the Maximum Entropy model (Sohl-Dickstein et al., 2011), that generated sparser undirected graphs. For the current study, such sparse graphs did not outperform PCC-based graphs (please see Supplementary material, Section 1). For undirected, weighted FC graphs (PCC, STTC), we trained Graph Convolutional Network (GCN; Kipf and Welling, 2016) and GraphSAGE models (Hamilton et al., 2017). For directed, unweighted FC graphs (CCH), Relational Graph Convolutional Networks (RGCNs; Schlichtkrull et al., 2018) were implemented to aggregate directed connectivity information with two types of edges (excitatory and inhibitory connections). As a result, we acquired a node embedding for each neuron that combines node features and connectivity defined by FC graphs.

### 3.2. Characterization of spontaneous network activity and single-neuron features

We performed *k*-means clustering on the UMAP-reduced single-neuron features to understand the variance between the neurons. The best number of clusters was *k* = 3, based on the silhouette score (Figure 2A). While one group showed narrower waveforms (smaller “Peak-to-trough,” “Trough-to-peak” values), the other two groups differed in the time constant, fitted to the downward slope in the auto-cross correlation histogram (“τ_decay_”) as shown in Figure 2B (see Section 2).

**Figure 3:**
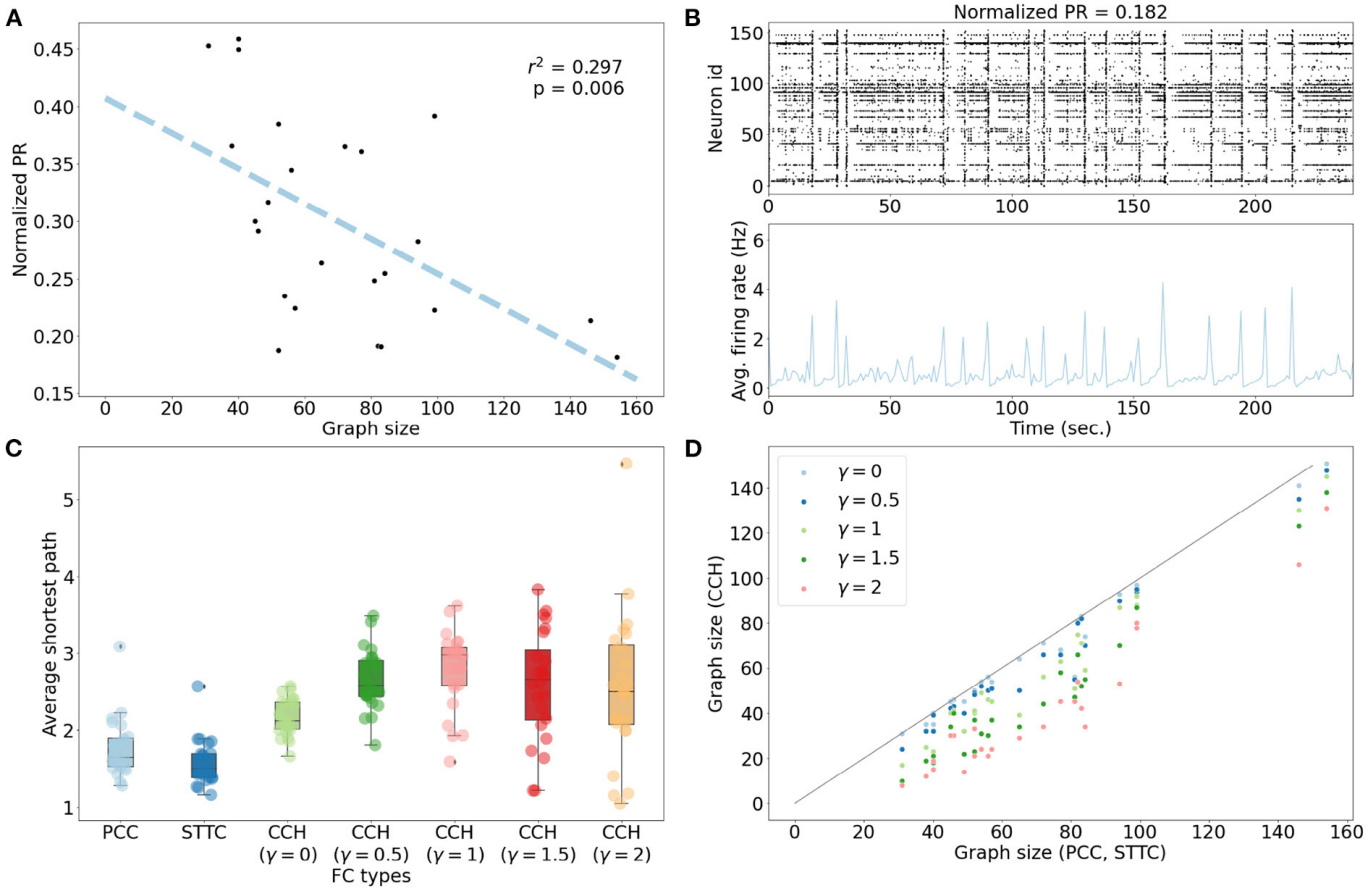
Characterization of spontaneous network activity. **(A)** All networks showed normalized *PR* values of <0.5, suggesting highly correlated network activity. There was a negative correlation between *PR* values and graph sizes (linear regression, *r*^2^ = 0.297, *p* = 0.006). **(B)** Network activity plots for the network with the lowest *PR* value. As depicted in the raster plot, the network showed clear synchronized firing activity. **(C)** The distributions of average shortest path for undirected FC graphs (PCC and STTC) revealed that most networks were close to fully-connected graphs featuring an average shortest path length of 1.765 ± 0.371 (μ ± σ) in PCC and 1.576 ± 0.288 in STTC. The directed FC graphs (CCH) showed longer average shortest paths: 2.162 ± 0.232 (γ = 0), 2.653 ± 0.39 (γ = 0.5), 2.784 ± 0.499 (γ = 1), 2.581 ± 0.704 (γ = 1.5), and 2.534 ± 0.943 (γ = 2). **(D)** The viable directed FC graphs derived from CCHs were smaller in comparison to the undirected FC graphs (PCC and STTC) for all γ values.

We then characterized the extent of correlated firing to understand the context for interpreting inferred FCs. If the network activity were uncorrelated with neurons firing independently of each other, then each edge in the FC graph would primarily reflect pairwise co-activity. However, if the network activity were highly correlated, each edge in the FC graph would also represent the effect of indirect interactions, such as synchronized firing. We measured how correlated the network activities were by computing Participation Ratios (PRs). The networks with a normalized *PR* value of <0.8 were considered to exhibit correlated network activity. All 24 networks indicated highly correlated network activity with a normalized *PR* value of <0.5 (Figure 3A). For example, the sub-network with the lowest normalized *PR* value shows clear synchronized firing activity (Figure 3B). We further investigated whether the variance in *PR* values resulted from the difference in the network sizes or in the average physical distances between neurons. There was a negative correlation between *PR* values and graph sizes (linear regression, *r*^2^ = 0.297, *p* = 0.006). This finding suggests that the required number of principal components to describe the variance in each network remained relatively stable [PR = 19.668 ± 6.861 (μ ± σ)] despite the differences in graph sizes [70.667 ± 30.986 (μ ± σ)]. There was no correlation between average physical distances between neurons and *PR* values (linear regression: *r*^2^ = 0.000, *p* = 0.946; Supplementary Figure 6).

Based on this correlated network activity, FC graphs were inferred from each network. As shown in Figure 3C, the distribution of the average shortest path between neurons had an average value of 1.765 ± 0.371 (μ ± σ) for PCC and 1.576 ± 0.288 (μ ± σ) for STTC, which suggested a high degree of inter-connectedness for undirected FCs (Supplementary Table 5). Upon comparing the edge weights of FC graphs, STTC FC graphs showed greater degree strengths than PCC FC graphs (Supplementary Figure 7). The directed graphs derived from CCH contained multiple disconnected graph components with small numbers of neurons. These disconnected components were not optimal for the GNN models, as these nodes cannot aggregate information from the neighborhood. As a result, these small components were discarded from the downstream analysis. The resulting directed FC graphs showed longer average shortest paths and smaller graph sizes compared to undirected FC graphs (PCC and STTC), as shown in Figures 3C, D.

### 3.3. Single-neuron and network firing rate responses to Bicuculline perturbation

**Figure 4:**
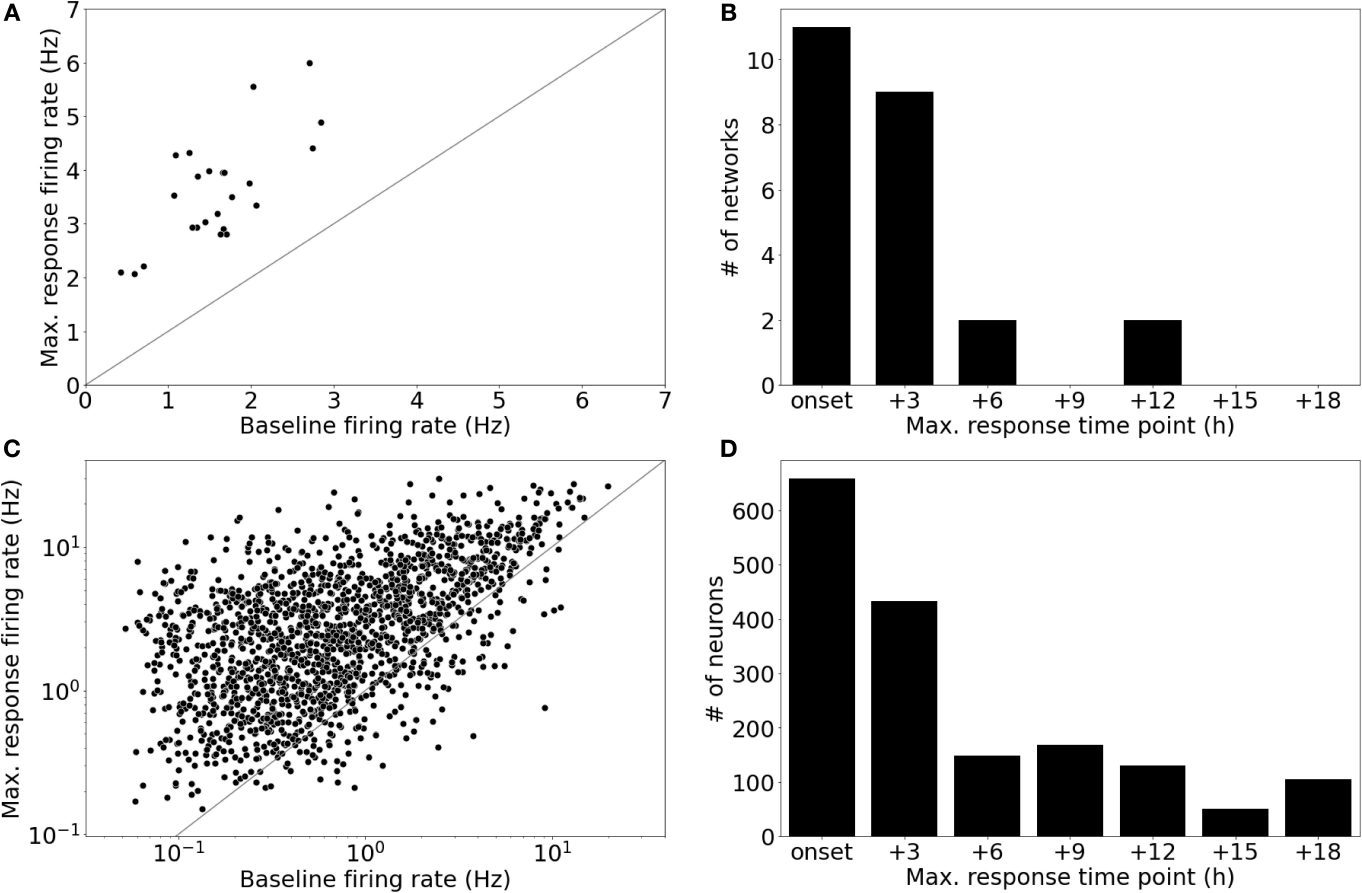
Single-neurons showed highly variable firing rate responses to Bicuculline perturbation. **(A)** All networks showed an increase in their population firing rate in response to Bicuculline (BIC, 5μM). The increase was measured in fold-change, Δ_*fch*_ [2.450 ± 0.827 (μ ± σ)]. **(B)** The majority of networks (*n* = 20/24) showed maximum firing rates within the first two time points after the perturbation (BIC, 5μM), while four networks showed maximum responses at later time points. **(C)** The pooled analysis of all neurons in the networks revealed that there was a subset of neurons (*n* = 146/1,695) that decreased their firing rates, while the network to which they belonged showed an increased firing rate. The differential fold-change, $\Delta_{fch^{\prime }}$, measured for each neuron showed a mean value of 5.9 ± 10.6 (μ ± σ). **(D)** Peaks in the firing rate responses were distributed over the entire perturbation time window. The neurons showed peaks in their firing rates even at time points where no network featured a peak in the firing rate.

All networks of the main dataset showed an increase in their population firing rate in response to BIC perturbation (5μM; Figure 4A). The network firing rate change of each network was measured in fold-changes ($\Delta_{fch}=\frac{\text{maximum FR}}{\text{baseline FR}}$). Averaged across all networks, the maximum response in population firing, measured across the entire perturbation period, was 2.450 ± 0.827 (μ ± σ). The majority of the recorded neuronal networks showed peaks in their network firing rates before 6 h following the onset of the perturbation (*n* = 20/24 networks); four networks showed activity peaks at 6 h or later time points (Figure 4B).

The pooled analysis of all neurons revealed that a subset of neurons showed decreased firing activity (*n* = 146/1695 neurons) compared to the baseline state (see Figure 4C). Due to this subset that decreased their activity, firing rate changes for neurons were measured in fold-change with respect to the difference in firing rates ($\Delta_{fch^{\prime }}=\frac{\text{maximum FR}-\text{baseline FR}}{\text{baseline FR}}$). The distribution of $\Delta_{fch^{\prime }}$ showed a mean value of 5.9 ± 10.6 (μ ± σ; Figure 4C). Interestingly, some neurons showed their maximum firing rate at later time points, where no network showed peaks in the firing rate (Figure 4D). Based on these results, we defined two prediction tasks: The first task was to predict the average firing rate during the two recording time points after the onset of BIC application (Task 1: immediate response). This task was motivated by looking at the distribution of peak timings of the network firing rates which were mostly located within this time window (6 h after onset). To account for neurons that showed maxima in their firing rates at later time points, the second task was to predict the maximum firing rate for each neuron during the entire perturbation window (i.e., across all seven recording time points, approx. 18 h; Task 2: maximum response).

### 3.4. Functional connectivity and single-neuron electrophysiological features were informative for predicting firing rate responses

Next, we trained a set of prediction models (GNN and non-GNN models) to understand whether the observed firing rate responses of individual neurons to BIC perturbation could be predicted by single-neuron electrophysiological features (node features) and inferred functional connectivity. We evaluated the models separately for undirected FCs (PCC and STTC) and directed FCs (CCH), as the two settings differed in the number of neurons. We denote node features and $\Delta_{fch^{\prime }}$ of a neuron *i* as **x**(*i*) and $\Delta_{fch^{\prime }}\left(i\right)$ respectively. Given a prediction model *f*(*x*), and the FC graph G that neuron *i* belongs to, the prediction task using GNN models can be written as an optimization of mean squared errors (MSEs) as follows:

$$\begin{array}{l} \text{minimize }\frac{1}{n}\overset{n}{\underset{i=1}{\sum }}{\left(f\left(\text{x}\left(i\right);G\right)-\Delta_{{\text{fch}}^{\prime }}\left(i\right)\right)}^{2}. \end{array}$$

To evaluate the impact of the structural information contributed by FC graphs, we trained linear regression and random forest regression models using only the single-neuron features (i.e., nodal features). All models were compared to the baseline model, which used the average of the target variables in the training dataset as the prediction value [Baseline prediction $=\frac{1}{n}\overset{n}{\underset{i=1}{\sum }}\Delta_{fch^{\prime }}\left(i\right),i\in \text{training dataset}$].

For each prediction model, we performed two-sided paired sample *t*-test using the MSEs of all neurons (nodes) to assess significance against the baseline model (α = 0.01). Across all networks, we observed that models without FC information (linear regression and random forest regression) showed average MSEs similar or worse to those of the baseline model (Table 1). Moreover, models using directed FC graphs (CCH) did not perform better than the baseline model (Supplementary Tables 7–11). We only observed significant improvement in the MSEs with the GraphSAGE models using undirected FC graphs.

**Table 1:** Performance comparison (average MSEs).

**Prediction models**	**FC type**	**Task 1. immediate response**	**Task 2. maximum response**
Baseline	N/A	1.083	1.069
Linear regression	N/A	1.090	1.099
Random forest regression	N/A	1.076 ± 0.001	1.091 ± 0.001
GraphSAGE-2-conv (max pooling)	PCC	0.992 ± 0.019 (*t*-test *p* = 0.002)	0.998 ± 0.018 (*t*-test *p* = 0.004)
**GraphSAGE-1-conv (max pooling)**	**PCC**	**0.991** **± 0.010 (*****t*****-test** ***p*** **<** **0.001)**	**0.993** **± 0.012 (*****t*****-test** ***p*** **=** **0.001)**

The table shows the average mean squared error (MSE) over all neurons (*n* = 1,695) for each prediction model. For models that have inherent stochasticity in the training step, a standard deviation is presented (30 runs). For linear regression and Baseline model, no standard deviation is shown as there was no stochasticity in the training step. Below the average MSE values that showed significant improvement (*p* < 0.01) compared to the average MSE of the baseline, *p*-values from two-sided paired *t*-test are presented. For both tasks, regression models that used only single-neuron features (linear regression and random forest regression) did not show improved performance over the baseline model. For both tasks, two GraphSAGE models based on PCC using max pooling with 1 convolutional layer (GraphSAGE-1-conv, max pooling, and PCC) and with two layers (GraphSAGE-2-conv, max pooling, and PCC) showed significantly better performance than the baseline model. The bold text here shows the best performing model with the lowest MSE value.

For both prediction tasks (Task 1: immediate response, Task 2: maximum response) we observed that two models based on PCC significantly outperformed the baseline model. The best model was the GraphSAGE model with one convolutional layer using max pooling (GraphSAGE-1-conv, max pooling, PCC). The second-best model was the GraphSAGE model with two convolutional layers using max pooling (GraphSAGE-2-conv, max pooling, PCC). This finding suggests that aggregating information through max pooling from the 1-hop (direct connection) and 2-hop neighbors (one node in between) was more generalizable than aggregating additional information from the 3-hop neighborhood. None of the GraphSAGE models with three convolutional layers (GraphSAGE-3-conv) showed an improvement over the baseline model performance (Supplementary Table 6). When looking at these networks separately, there were few networks that showed worse network-averaged MSEs compared to those of the baseline model even for the best model (Supplementary Figure 8).

#### 3.4.1. Validating the effect of single-neuron/FC features on improved prediction performance

We then probed the best performing GNN model (GraphSAGE-1-conv, max pooling, PCC) to test whether the improvements in MSEs were attributable to the experimental values, such as node features and inferred PCC FCs. First, we tested the contribution of PCC FCs by generating randomized PCC FC graphs. Briefly, we generated fully-connected graphs with each edge-weight randomly sampled from a uniform distribution in the range [0, 1] (“Random sampled”). Additionally, we tested two versions of shuffled PCC FC graphs by (1) shuffling all edges in the graph (“PCC shuffled”) and (2) shuffling edges while preserving node degrees (“PCC shuffled deg. preserved”). As shown in the Table 2, predictions on networks with randomized connectivity resulted in larger average MSEs than the MSEs of the best model (two-sided *t*-test, *p* < 0.001).

**Table 2:** Ablation study (average MSEs).

**Condition**	**FC type**	**Node features**	**Task 1. immediate response**	**Task 2. maximum response**
**Best model**	**PCC**	**Experimental**	**0.991** **±0.010**	**0.993** **±0.012**
Randomized FC	Random sampled	Experimental	1.082 ± 0.009	1.103 ± 0.016
Randomized FC	PCC shuffled	Experimental	1.068 ± 0.006	1.101 ± 0.016
Randomized FC	PCC shuffled deg. preserved	Experimental	1.062 ± 0.007	1.062 ± 0.014
Sampled FC	PCC min. spanning tree	Experimental	1.058 ± 0.005	1.070 ± 0.007
Altered node features	PCC	Avg. of target variables	1.055 ± 0.002	1.027 ± 0.002
Altered node features	PCC	Random sampled	1.076 ± 0.005	1.051 ± 0.006
Altered node features	PCC	Random shuffled	1.067 ± 0.007	1.034 ± 0.007

The table shows average MSEs (*n* = 1,695) resulting from different variants of the best performing model (GraphSAGE-1-conv, max pooling, and PCC). To test the contribution of connectivity information in the form of PCC FC graphs, the model (GraphSAGE-1-conv and max pooling) was trained on three randomized and one sampled PCC FC variants. For randomized variants, we generated fully-connected graphs with edge weights randomly sampled from the uniform distribution in the range [0, 1] (“Random sampled”). Then, we further generated randomized variants by shuffling (1) all edges in the PCC FC graphs (“PCC shuffled”) and (2) shuffling edges but keeping node degree strengths (“PCC shuffled deg. preserved”). We also computed minimum spanning trees from the PCC FC graphs (“PCC min. spanning tree”) to sample strongly connected edges. All of these variants showed worse MSEs compared to the best model, for which experimental PCC FC graphs were used. Next, we tested the contribution of node features by training two additional cases with altered node features. We trained a model with the experimental PCC FC graphs but swapped the node features with the average of target variables (‘Avg. of target variables”). Then a model was trained with randomly sampled node features from the uniform distribution in the range [0,1] (“Random sampled”). Finally, we trained a model by randomly shuffling each feature among nodes (“Random shuffled”). For all cases, the resulting average MSEs were worse than those of the best model, which used experimental node features. The bold text here shows the best performing model with the lowest MSE value.

As stated earlier, results indicated that the max pooling operation resulted in better prediction accuracy (lower MSEs) compared to the mean pooling operation (Supplementary Table 6, Supplementary Figures 9, 10). To test if this performance improvement could be explained by a back-bone of strongly-connected edges in the biological networks, we inferred minimum spanning trees (Kruskal, 1956) from PCC FC graphs (“PCC min. spanning tree”). However, for both tasks, the minimum spanning tree-derived graphs, showed larger MSEs compared to the values obtained for the best model (Table 2; *t*-test, *p* < 0.001).

Finally, we tested the contribution of HD-MEA inferred single-neuron/nodal features to the prediction accuracy of GNNs, while keeping the experimental PCC FC graphs the same (as experimentally observed). This time, we used the average of the target variables as node features, which was a GNN extension of the baseline model. Results indicated that the average MSEs for both tasks were higher than those of the best model using experimental values (Table 2), however, with less statistical significance (Task 1, *p* = 0.005, Task 2, *p* = 0.119). In addition, we swapped each element in the node feature vectors with randomly sampled values from the uniform distribution in the range [0, 1]. The randomization of node features again resulted in larger average MSEs (Table 2; Task 1: *p* < 0.001, Task 2: *p* = 0.008). Finally, we randomly shuffled each feature among nodes to train a model. This shuffling again resulted in worse MSEs (Table 2; Task 1: *p* < 0.001, Task 2: *p* = 0.039). From these two tests and our previous results of models using only single-neuron/nodal features (linear regression, random forest regression), we concluded that nodal features may only be useful for the prediction when combined with FCs.

#### 3.4.2. Generalization to additional test datasets

**Figure 5:**
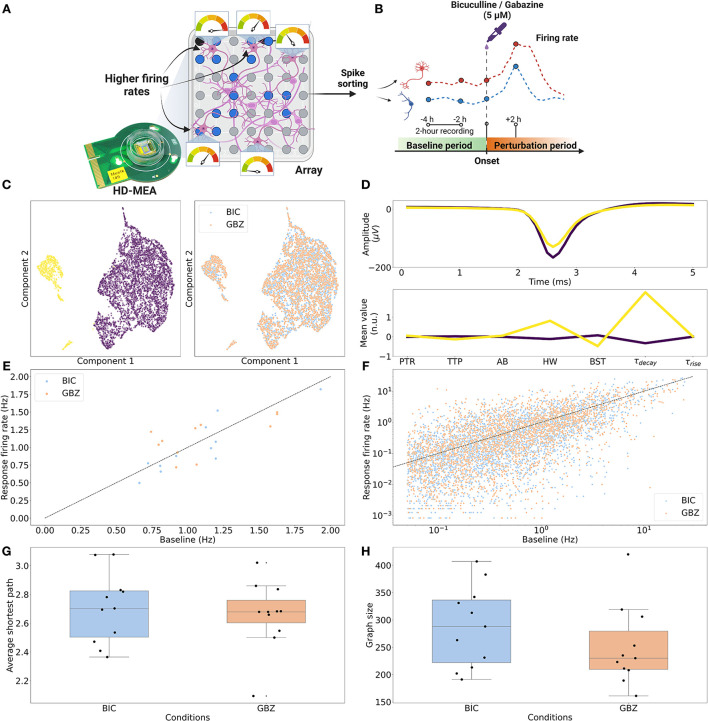
Firing rate changes and graph characterization of the test dataset. **(A)** HD-MEA recordings were acquired prioritizing electrodes detecting higher firing rates across the entire sensing area. **(B)** Gabazine (GBZ) (5μM; 11 recordings) and Bicuculline (BIC) (5μM; 11 recordings) were applied to measure immediate perturbation responses (Task 1. <+6 h after the onset). **(C)** (Left) UMAP-reduced single-neuron features were best clustered (*k*-means clustering) with *k* = 2 based on the silhouette score. (Right) Between two perturbation conditions, there was no obvious difference in the distribution of the features. **(D)** (Top) The averaged waveform for each cluster. (Bottom) Between the two clusters, τ_decay_ showed the largest difference in the average normalized (standard scaled) value. **(E)** The application of perturbation (BIC and GBZ) resulted in both increase and decrease in population firing rates. Fold-changes (Δ_*fch*_) in population firing rates for BIC showed the mean of 0.932 ± 0.157 (μ ± σ) and 1.072 ± 0.273 for GBZ. **(F)** Differential fold-change of single-neuron firing rates ($\Delta_{fch^{\prime }}$) showed a large variance with the mean of 0.247 ± 2.233 and 0.597 ± 4.132 for BIC and GBZ, respectively. **(G)** PCC FC graphs derived from these networks exhibited average shortest paths of 2.711 ± 0.244 (BIC) and 2.669 ± 0.242 (GBZ). **(H)** Networks contained on average 289 ± 74.318 neurons for BIC and 253.455 ± 76.764 neurons for GBZ.

We further tested the generalizability of the above GraphSAGE results by applying the same analysis pipeline to HD-MEA recordings acquired under altered experimental conditions. We relaxed the recording condition to record from the entire HD-MEA sensing area rather than from dense electrode configurations (see Methods). We acquired additional perturbation recordings by using Gabazine (GBZ) (5μM), another GABA_*A*_ receptor antagonist, as well as BIC. In total, 22 recordings were tested: 11 recordings were perturbed with BIC (5μM), and 11 recordings were perturbed with GBZ (5μM). For this dataset, only immediate responses (Task 1: <+6 h after the onset) were available (Figures 5A, B). Single-neuron features of all neurons for both conditions (BIC and GBZ; *n* = 5,919) were pooled for a clustering analysis. UMAP-reduced embeddings were best clustered into two clusters based on the silhouette score (Figure 5C left). There was no clear difference in the distribution of single-neuron features between the two perturbation conditions (Figure 5C right). The most important difference between the two clusters was apparent for τ_decay_ upon comparing the mean value of single-neuron features (Figure 5D). In response to the compound perturbation, recordings showed both increases and decreases in population firing rate changes, and large variance in the firing rate responses of individual neurons (Figures 5E, F). The differential firing rate fold-changes ($\Delta_{fch^{\prime }}$) of the neurons featured mean values of 0.247 ± 2.233 (μ ± σ) (*n* = 3,164) and 0.597 ± 4.132 (*n* = 2,755) for BIC and GBZ, respectively. As for the main dataset, PCC FC was inferred for each recording. The inferred FC graphs showed longer average shortest paths compared to the FCs in the main experiment (1.765 ± 0.371) with 2.711 ± 0.244 (BIC) and 2.669 ± 0.242 (GBZ) (Figure 5G). Each network contained more neurons compared to the main experiment (70.667 ± 30.986) with 289 ± 74.318 neurons (BIC) and 253.455 ± 76.764 neurons (GBZ; Figure 5H).

GraphSAGE models with max pooling again showed better accuracy compared to the mean pooling variants and the non-GNN models for BIC perturbation (Supplementary Table 12). In contrast to the main experiment where 1-layer variant was the best model, the 3-layer variant (GraphSAGE-3-conv and max pooling) showed the best performance (Table 3). Interestingly, none of the GraphSAGE models showed significantly improved performance for the networks perturbed with GBZ although the 2-layer GraphSAGE model with max pooling (GraphSAGE-2-conv and max pooling) showed the best performance for the GBZ condition (Supplementary Table 12).

**Table 3:** Performance comparison (test dataset and average MSEs).

**Prediction models**	**FC type**	**Bicuculline (*n* = 3,164)**	**Gabazine (*n* = 2,755)**
Baseline	N/A	1.031	1.267
Linear regression	N/A	1.018 (*t*-test *p* = 0.004)	1.260
Random forest regression	N/A	1.034 ± 0.001	1.261 ± 0.001
GraphSAGE-2-conv (max pooling)	PCC	0.985 ± 0.006 (*t*-test *p* < 0.001)	**1.257** **± 0.004**
GraphSAGE-3-conv (max pooling)	PCC	**0.979** **± 0.006 (*****t*****-test** ***p*** **<** **0.001)**	1.269 ± 0.010

The table shows average MSEs (*n* = 3,164, BIC; *n* = 2,755, GBZ) of GraphSAGE models to predict firing rate changes of neurons. For models that showed significant improvement (*p* < 0.01) compared to the average MSE of the baseline, *p*-values from two-sided paired *t*-test are presented. For BIC perturbation, a 3-layer GraphSAGE model with max pooling showed the best prediction accuracy. None of the GraphSAGE models showed an improvement in prediction accuracy that was statistically significant for the networks perturbed with GBZ. The 2-layer GraphSAGE model with max pooling operation showed the best prediction accuracy for GBZ perturbation. The bold texts here show the lowest MSE value for each experimental dataset (Bicuculline, Gabazine).

## 4. Discussion

The aim of our study was to assess the potential of GNNs to predict firing rate changes of neurons under pharmacological perturbation. We hypothesized that joint representations of single-neuron electrophysiological features/nodal features and functional connectivity information, generated by GNN models, could show improved prediction accuracy compared to models that do not include information on the underlying functional connectivity between neurons. We addressed this question by perturbing primary rodent hippocampal neurons with BIC (5μM) and by trying to predict firing rate changes of individual neurons using GNNs and classical machine learning models. We found that firing-rate responses ($\Delta_{fch^{\prime }}$) of neurons exhibited greater variance compared to the network firing rate responses (Δ_*fch*_) to perturbations. We showed that GNN-generated joint representations of extracellular features and FC yielded moderate, yet statistically significant, improvements in predictions of single-neuron firing rate responses. We confirmed that the extracellular features alone did not yield good predictions of the perturbation responses in the main experiment. Yet, the interpretation of this result requires further considerations. First, extractions of extracellular electrophysiological features are known to be susceptible to experimental conditions and recording modalities. Previous *in vivo* studies reported that extracellular electrophysiological features extracted by means of recording electrodes could be useful indicators for cell-type classification (Senzai et al., 2019; Mosher et al., 2020; Petersen and Buzsáki, 2020), whereas Weir et al. (2014) reported that extracellular electrophysiological features *in vitro* were not indicative of excitatory and inhibitory neurons. Therefore, we want to emphasize that the current study only addresses the usefulness of employing extracellular features for the prediction task at hand, but does not provide information on the behavior or nature of different cell types undergoing the perturbation. Other methods to extract extracellular features could help to address this issue. For example, Lee et al. (2021) recently demonstrated the advantage of using non-linear dimensionality-reduction techniques on extracellular waveforms to distinguish functionally distinct neurons *in vivo*. Future studies providing ground truths for such approaches could enable a more reliable extraction of extracellular features. In addition, recent fluorescence microscopy techniques could be combined with HD-MEA data to include additional node features including subcellular neural dynamics, such as axonal and dendritic dynamics (Cornejo et al., 2022; Kim and Schnitzer, 2022).

The prediction assessment of undirected FC methods (PCC and STTC) showed that the models trained on PCC-derived FC graphs yielded better prediction performance (Supplementary Table 6). We hypothesize that the inclusion of inactive periods between spike trains could provide a better estimate for the correlation when predicting the perturbation responses of neurons. Although the comparison of models trained on undirected FCs is fair, we need to be cautious when comparing models trained with different directed FCs or models using directed vs. undirected FCs. In the case of directed FCs, we explored the parameter γ to train models on networks of different sizes. Due to the number of nodes being different and the fact that the GNN models generalize better with more samples, we cannot make a fair comparison of these models' predictive performance. The same would apply if we attempted to compare predictions between models trained on undirected vs. directed networks. However, in order to gain insights into the limitations of models relying on directed FCs, we analyzed the effects of the sparsity in the connectivity network and the reduction in the number of training neurons (Supplementary Table 5 and Section 2 in Supplementary material). We speculate that these two factors might have harmed the generalizability of GNN models as the information passing was limited in these directed FCs (Supplementary Tables 7–11).

When comparing GCN and GraphSAGE models, GraphSAGE models attained the best performance (Supplementary Figures 9, 10). The pooling operation of GraphSAGE models resulted in enhanced performance compared to the GCN models. When comparing two types of pooling operations, the max pooling operation showed superior performance in comparison to the mean pooling operation. Interestingly, we observed that the max pooling model with 1 and 2 convolutional layer(s) performed better than the 3-layer variants. This result was in agreement with the distribution of average shortest paths of undirected FCs, reported earlier, where most values were <2. Moreover, the training-validation gap of the 3-layer variants suggested that there was a clear overfitting, and that these deeper GNN models may require more data to perform better. When looking at each network separately, there were networks that showed worse MSEs, compared to the baseline, even for the best performing GraphSAGE method. We observed that one of these networks showed clear signs of overfitting despite the implemented training measure (dropout layer), but we could not observe similar behavior for the remaining networks (Supplementary Figures 11, 12). From a post-hoc analysis of MSEs obtained from the best GraphSAGE model, we found that a subset of neurons showed large firing rate changes ($\Delta_{fch^{\prime }}$), accompanied by large MSEs (Supplementary Figure 13). The distribution of MSEs was heavily skewed recapitulating the skewed distribution of target variables (Supplementary Figure 14). This suggested that more data on the respective outliers would be needed for the algorithm to learn their responses more reliably.

To test the generalizability of the result, GraphSAGE models were further applied to HD-MEA recordings using electrode configurations that prioritized electrodes with high firing-rates. For both perturbations (BIC, GBZ), we again observed the best prediction accuracy with GraphSAGE models. This finding suggests that, even upon sampling neurons prioritizing firing rates rather than physical vicinity, the joint representation of single-neuron electrophysiological features and inferred functional connectivity *via* GNNs was more predictive than using them individually. Yet, there were important observations for each perturbation condition that differed from the result of the main experiment. The networks that underwent BIC perturbation showed best prediction performance of the 3-layer GraphSAGE max pooling model as opposed to the main experiment, where the 1-layer variant was the best model. This result suggests that extracting higher-order information beyond direct neighbors could be crucial for more accurate prediction. We also probed the networks that were perturbed with GBZ. Surprisingly, while the 2-layer GraphSAGE model with max pooling showed marginally improved accuracy in comparison to the baseline model, none of the GraphSAGE models performed significantly better. Previous studies have indicated that although BIC and GBZ are both considered GABA_*A*_ receptor antagonists, BIC is known to additionally affect Ca^2+^ dependent K^+^ currents in contrast to GBZ (Johansson et al., 2001; Paul et al., 2003). We hypothesize that the max pooling operation of GraphSAGE models could have captured the higher-order interactions arising from the Ca^2+^-activated depolarizing K^+^ currents.

Our findings support the application of GraphSAGE-like GNNs with inductive modeling capability to capture complex interactions between neurons as a “black-box” solution to model responses of neurons. The potential of GNN models to explain neural activity is, in our view, largely under-explored and poses future challenges. First, the learned GNN models need to be analyzed with matching explainer methods to interpret the inner workings of the model. Recent advances in explainer methods enabled the extraction of simplified relations between input features and target variables in complex GNN models. Although methods vary in how they extract prominent information from the joint representation of graph structures and node features, they yielded convincing explanations for graph/node classification tasks by detecting meaningful graph motifs, molecular functional groups and image pixel locations (Pope et al., 2019; Ying et al., 2019; Rex et al., 2020; Schnake et al., 2021). In addition, the prediction accuracy of the models, presented in this study shows that we are still far from achieving full modeling of neuronal dynamics. Further developments in recording modalities to capture more neurons and the use of more expressive GNN models (Battaglia et al., 2016; Cranmer et al., 2020, 2021) could enhance the accuracy of such modeling attempts. In combination with advancements in interpretable graph learning, we expect a self-reinforcing cycle that could deepen our understanding of neural circuits.

In conclusion, we found that the inductive GNN model (GraphSAGE) generated joint representations of single-neuron/nodal features and FCs, which improved predictions of firing-rate changes of neurons upon pharmacological perturbation. Our findings could be applied to a broad range of neuroscientific studies utilizing microelectrode-array recordings of extracellular electrical activity.

## Data availability statement

The original contributions presented in the study are publicly available. This data can be found at: https://github.com/arahangua/gnn_prediction_sn.

## Author contributions

TK, DC, PH, MS, and DR contributed to conception and design of the study. AH, DC, MS, and DR provided supervision to TK throughout the study. JB, VE, and SR performed the dissection of rat embryos to obtain primary neuronal cultures. AH provided necessary resources and materials for the current study. TK performed the experiment and analysis and wrote the first draft of the manuscript. All authors contributed to manuscript revision, read, and approved the submitted version.
