# Supplementary material for: Predicting in vitro single-neuron firing rates upon pharmacological perturbation using Graph Neural Networks
Source: Front Neuroinform. 2023 Jan 11;16:1032538. doi: 10.3389/fninf.2022.1032538 (PMC9874697; doi:10.3389/fninf.2022.1032538)
Supplement: Supplementary file 1 [file Data_Sheet_1.pdf]

Supplementary Material

Table S1. Parameters for spike-sorting (Kilosort2)

**Parameters**	value
ops.fs	20000
ops.fshigh	150
ops.minfr_goodchannels	0
ops.Th	[10 4]
ops.lam	10
ops.AUCsplit	0.9
ops.minFR	1/50
ops.momentum	[20 400]
ops.sigmaMask	30
ops.ThPre	8
ops.spkTh	-6
ops.reorder	1
ops.nskip	25
ops.nfilt_factor	4
ops.ntbuff	64
ops.NT	4*64*1024+ ops.ntbuff
ops.whiteningRange	32
ops.nSkipCov	25
ops.scaleproc	200
ops.nPCs	3

Table S2. Silhouette score for different number of clusters

Number of clusters (k)	Main dataset	Test dataset
2	0.601	**0.585**
3	**0.617**	0.432
4	0.472	0.439
5	0.502	0.434
6	0.498	0.438
7	0.491	0.452
8	0.474	0.464
9	0.475	0.447
10	0.482	0.453
11	0.466	0.446
12	0.421	0.430

Table S3. Grid search parameters for random forest regressor

**Parameters**	Range
Number of trees in models	np.linspace(start = 100, stop = 1000, num = 10)
Number of features used	‘sqrt’, ‘all’
Required number of samples for splitting	2, 4, 6, 8, 10
Required number of samples for each node	1, 2, 3, 4, 5

Table S4. Grid search parameters for GNN models

**Parameters**	Range
dropout probability	0.1, 0.2, 0.3, 0.4
learning rates (ADAM)	0.001, 0.005, 0.01
l2 regularization (ADAM)	0.0001, 0.001, 0.01
number of hidden dimensions	8, 16, 32
epoch	[1, 1000]

Table S5. Average shortest paths The table shows average shortest path lengths for each network along with the number of neurons considered for the training/testing of GNN models. For directed graphs, average shortest paths for different γ values are presented.

Network id	PCC (n=1695)	STTC (n=1695)	CCH ($\gamma = 0$) ($n = 1616$)	CCH ($\gamma = 0.5$) ($n = 1509$	CCH ($\gamma = 1$) ($n = 1290$)	CCH ($\gamma = 1.5$) ($n = 1070$)	CCH ($\gamma = 2$) ($n = 795$)
0	1.528	1.461	2.062	2.863	3.151	1.212	1.043
1	1.563	1.495	2.317	2.413	2.959	2.592	3.172
2	2.100	1.851	1.883	2.452	2.337	2.603	2.505
3	3.083	2.564	1.658	1.803	1.928	2.283	2.576
4	1.322	1.249	2.533	2.483	3.037	3.456	1.991
5	1.594	1.425	2.33	3.484	2.540	1.73	1.172
6	1.275	1.156	2.369	2.936	3.614	2.764	1.993
7	1.479	1.383	2.129	2.719	3.012	3.546	3.372
8	1.511	1.422	2.357	3.41	3.048	2.443	2.093
9	1.426	1.262	2.205	2.629	2.999	3.32	2.446
10	1.850	1.691	1.852	2.157	2.590	3.276	3.307
11	2.059	1.882	1.975	2.349	2.806	2.742	2.606
12	1.825	1.662	1.894	2.146	2.583	2.953	3.771
13	1.606	1.442	2.565	2.941	1.591	1.212	1.147
14	1.786	1.598	2.394	2.891	3.251	2.062	2.11
15	1.908	1.695	2.111	2.867	3.540	3.499	3.256
16	1.793	1.591	2.188	3.042	2.991	2.696	2.485
17	1.884	1.676	2.035	2.522	2.798	2.752	2.851
18	2.146	1.881	1.999	2.476	3.124	3.83	5.466
19	1.658	1.511	2.057	2.791	2.995	2.427	2.69
20	1.509	1.347	2.402	2.437	3.141	2.144	2.144
21	2.217	1.832	2.003	2.445	2.799	2.89	3.08
22	1.634	1.383	2.496	3.104	2.053	1.636	1.4
23	1.609	1.365	2.07	2.313	1.921	1.887	2.139

Table S6. Average MSE (n=1695, undirected FC)

**Models**	Task 1. Immediate response	Task 2. Maximum response
Baseline	1.083	1.069
Linear regression	1.091	1.099
Random forest regression	1.076 ± 0.001	1.091 ± 0.001
GraphSAGE-1-conv (max pooling, STTC)	1.019 ± 0.011	1.034 ± 0.008
GraphSAGE-2-conv (max pooling, STTC)	1.043 ± 0.020	1.033 ± 0.022
GraphSAGE-3-conv (max pooling, STTC)	1.038 ± 0.021	1.056 ± 0.018
GraphSAGE-1-conv (mean pooling, STTC)	1.065 ± 0.011	1.074 ± 0.005
GraphSAGE-2-conv (mean pooling, STTC)	1.078 ± 0.015	1.073 ± 0.019
GraphSAGE-3-conv (mean pooling, STTC)	1.077 ± 0.012	1.057 ± 0.013
GCN-1-conv (STTC)	1.110 ± 0.040	1.089 ± 0.008
GCN-2-conv (STTC)	1.102 ± 0.027	1.125 ± 0.017
GCN-3-conv (STTC)	1.098 ± 0.031	1.168 ± 0.037
GraphSAGE-1-conv (max pooling, PCC)	0.991 ± 0.011	0.993 ± 0.012
GraphSAGE-2-conv (max pooling, PCC)	0.992 ± 0.019	0.998 ± 0.018
GraphSAGE-3-conv (max pooling, PCC)	1.006 ± 0.026	1.048 ± 0.037
GraphSAGE-1-conv (mean pooling, PCC)	1.061 ± 0.007	1.100 ± 0.031
GraphSAGE-2-conv (mean pooling, PCC)	1.036 ± 0.016	1.039 ± 0.013
GraphSAGE-3-conv (mean pooling, PCC)	1.079 ± 0.022	1.041 ± 0.014
GCN-1-conv (PCC)	1.116 ± 0.040	1.102 ± 0.017
GCN-2-conv (PCC)	1.084 ± 0.027	1.112 ± 0.025
GCN-3-conv (PCC)	1.112 ± 0.032	1.177 ± 0.080
GraphSAGE-1-conv (max pooling, Random sampled)	1.082 ± 0.009	1.103 ± 0.016
GraphSAGE-1-conv (max pooling, PCC shuffled)	1.068 ± 0.006	1.101 ± 0.016
GraphSAGE-1-conv (max pooling, PCC shuffled deg. preserved)	1.062 ± 0.007	1.062 ± 0.014
GraphSAGE-1-conv (max pooling, PCC min. spanning tree)	1.058 ± 0.005	1.070 ± 0.007

Table S7. Average MSE ($\gamma = 0$, n=1616, directed FC)

**Models**	Task 1. Immediate response
Baseline	1.09
RGCN-1-conv	1.083 ± 0.006
RGCN-2-conv	1.082 ± 0.009
RGCN-3-conv	1.089 ± 0.01

Table S8. Average MSE ($\gamma = 0.5$, n=1509, directed FC)

**Models**	Task 1. Immediate response
Baseline	1.123
RGCN-1-conv	1.127 ± 0.01
RGCN-2-conv	1.167 ± 0.022
RGCN-3-conv	1.151 ± 0.016

Table S9. Average MSE ($\gamma = 1$, n=1290, directed FC)

**Models**	Task 1. Immediate response
Baseline	1.237
RGCN-1-conv	1.248 ± 0.01
RGCN-2-conv	1.232 ± 0.015
RGCN-3-conv	1.213 ± 0.021

Table S10. Average MSE ($\gamma = 1.5$, n=1070, directed FC)

**Models**	Task 1. Immediate response
Baseline	1.253
RGCN-1-conv	1.261 ± 0.015
RGCN-2-conv	1.317 ± 0.023
RGCN-3-conv	1.326 ± 0.023

Table S11. Average MSE ($\gamma = 2$, n=795, directed FC)

**Models**	Task 1. Immediate response
Baseline	1.678
RGCN-1-conv	1.654 ± 0.015
RGCN-2-conv	1.826 ± 0.031
RGCN-3-conv	1.7 ± 0.027

Table S12. Performance comparison (test dataset, average MSEs)

**Prediction models**	FC type	Bicuculline (n=3164)	Gabazine (n=2755)
Baseline	N/A	1.031	1.267
Linear regression	N/A	1.018	1.260
Random forest regression	N/A	1.034 ± 0.001	1.261 ± 0.001
GraphSAGE-1-conv (max pooling)	PCC	0.999 ± 0.002	1.268 ± 0.011
GraphSAGE-2-conv (max pooling)	PCC	0.985 ± 0.006	1.257 ± 0.004
GraphSAGE-3-conv (max pooling)	PCC	0.979 ± 0.006	1.269 ± 0.010
GraphSAGE-1-conv (mean pooling)	PCC	0.992 ± 0.004	1.262 ± 0.004
GraphSAGE-2-conv (mean pooling)	PCC	0.994 ± 0.004	1.287 ± 0.021
GraphSAGE-3-conv (mean pooling)	PCC	1.006 ± 0.011	1.279 ± 0.039

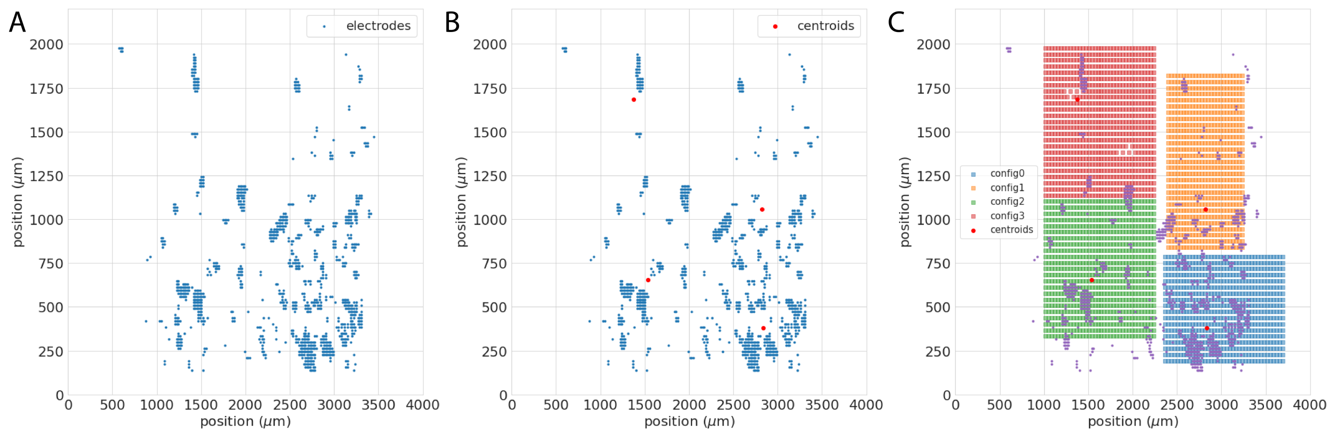

Figure S1. Generation of dense recording configurations (A) From the most active electrodes (prioritizing firing rates, up to 1024 electrodes), (B) *k*-means centroids (*k*=4) were generated. (C) Four dense-recording configurations were generated manually using the generated centroids as reference points to include most active electrodes.

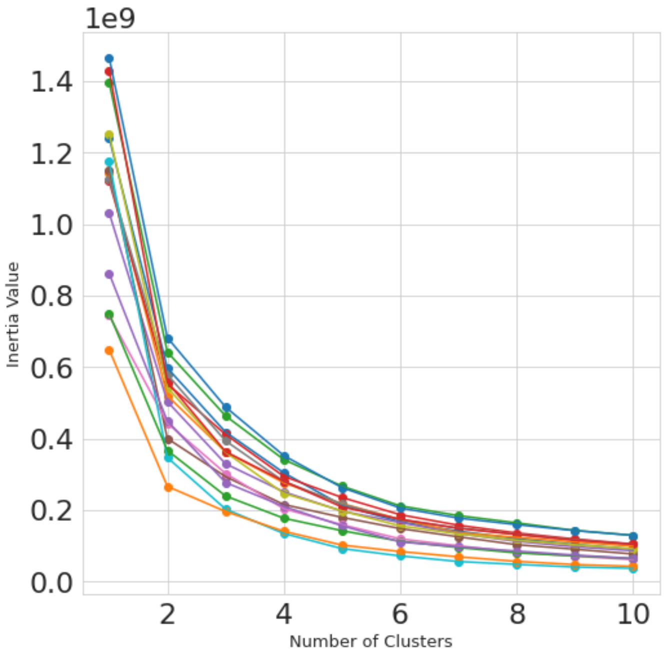

Figure S2. Curve of inertia Based on these inertia value curves, we chose K=4 as it was the optimal point where the intra-cluster distances between most active electrodes were small enough with a relatively small number of clusters.

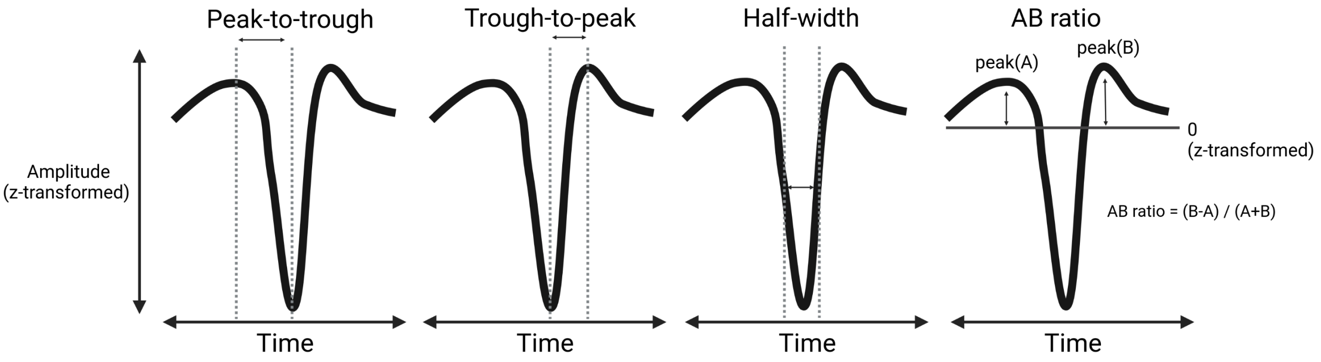

Figure S3. **Waveform features**

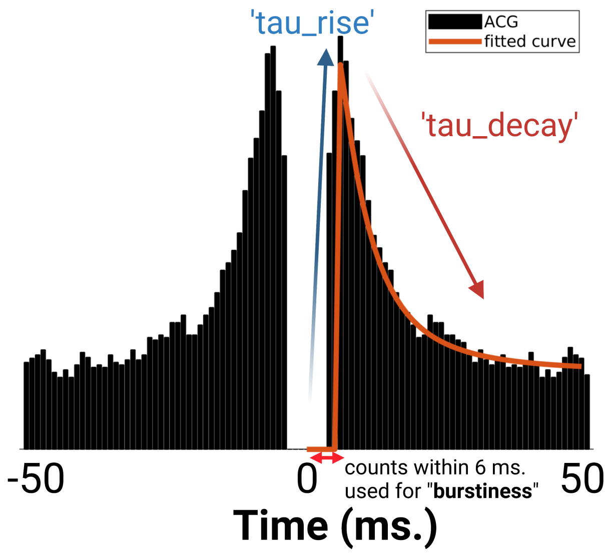

Figure S4. **Firing pattern features**

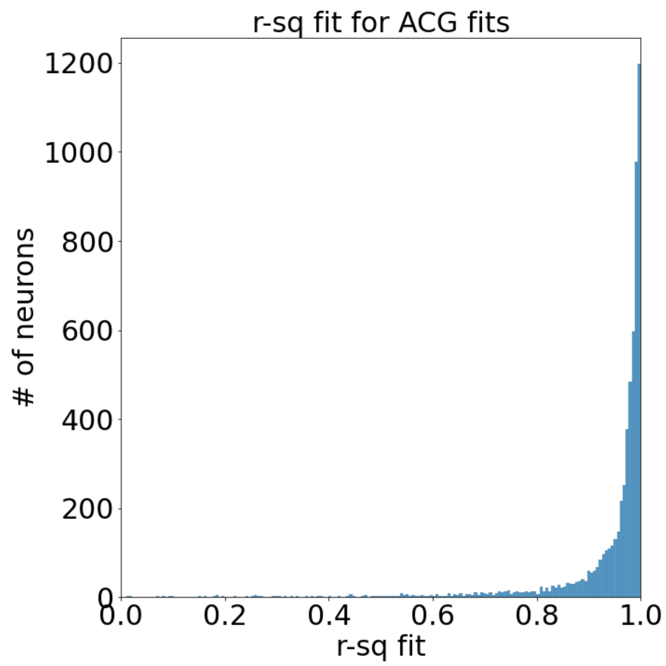

Figure S5. R-square values of ACG fits Given the distribution of r-square values, neurons showing r-square fits lower than 0.8 were discarded.

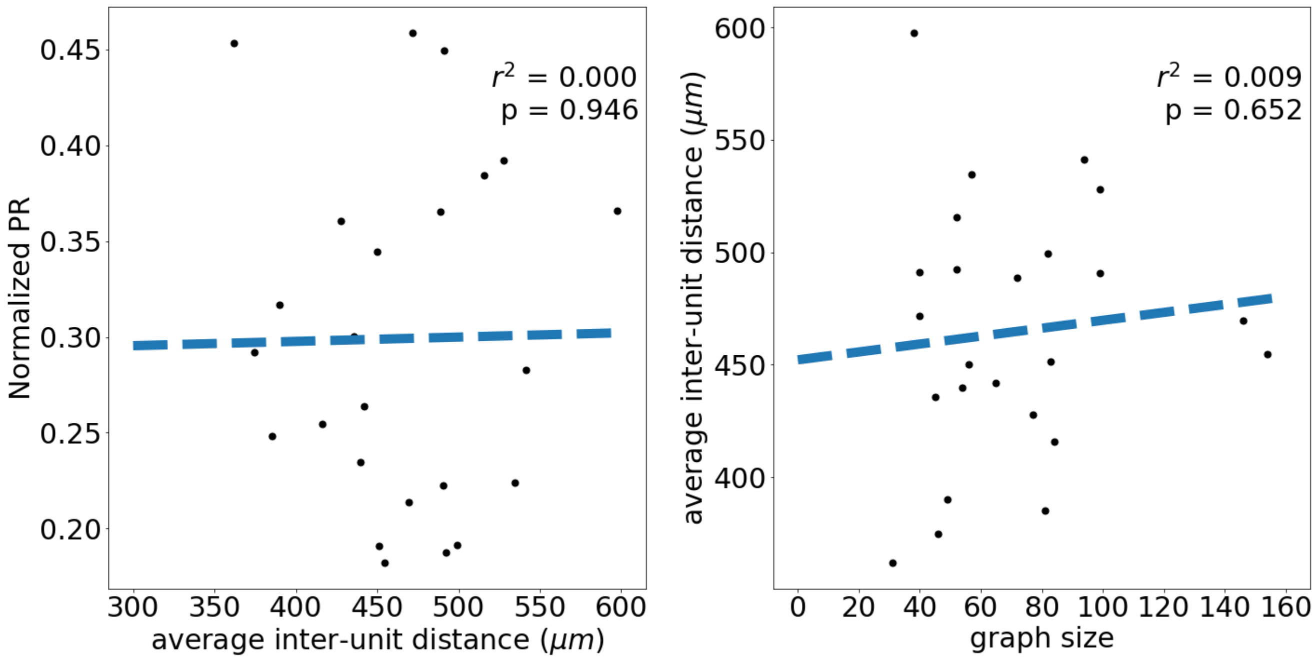

Figure S6. Culture condition dependent factors For the average physical distance between neurons and normalized PR values, we could not observe any significant correlation. Similarly, the image does not show any significant correlation between the average physical distance between neurons and normalized PR values.

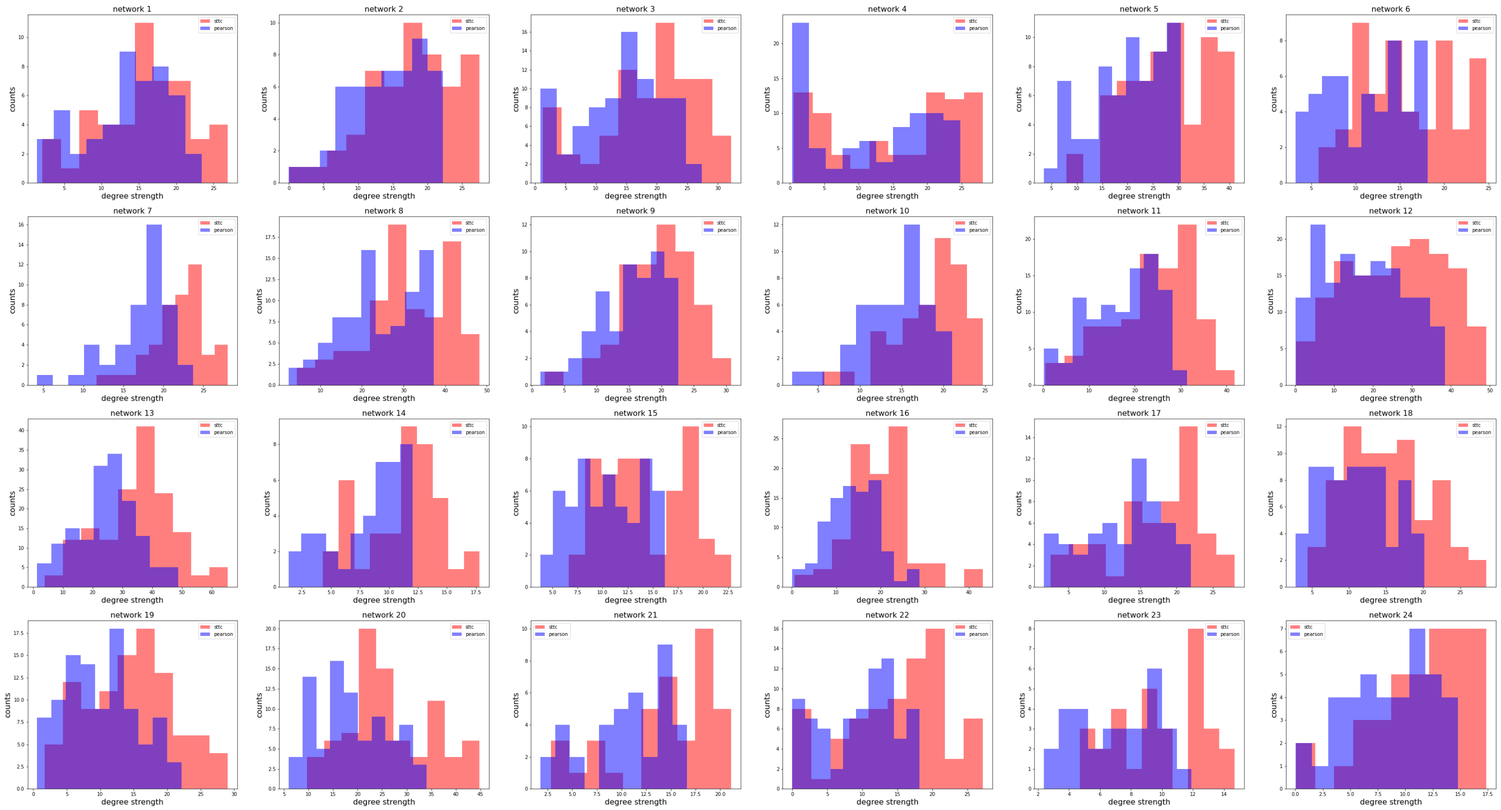

Figure S7. Distribution of the degree strength for each network The figure shows the degree strength distribution of each network. For all networks, the degree strength measured in STTCs showed larger values than measured in PCC.

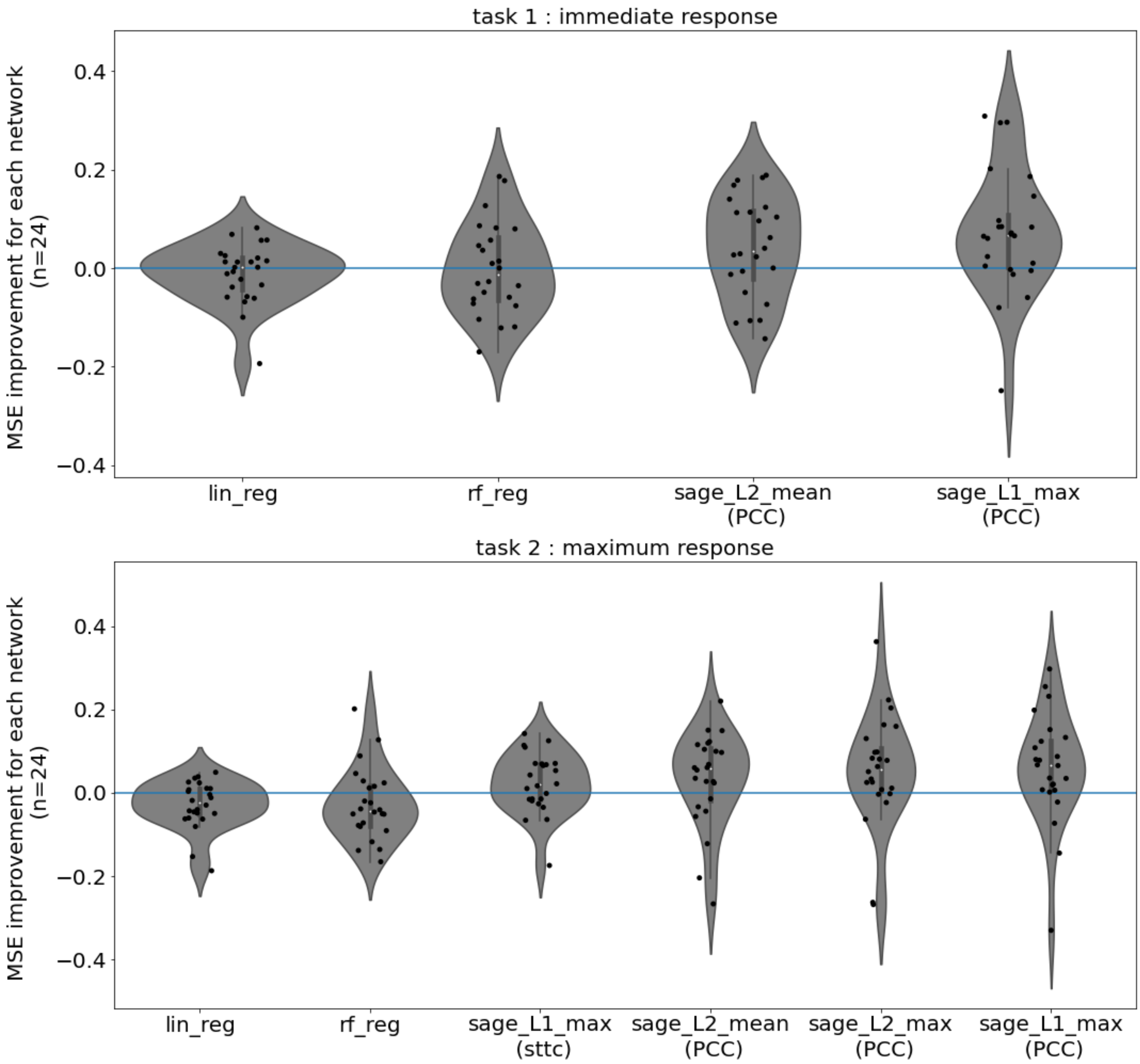

Figure S8. Distribution of MSE improvements for each network The figure shows MSE improvements compared to the baseline model (blue line). Each data point in the violin plots corresponds to the MSE improvement of a network.

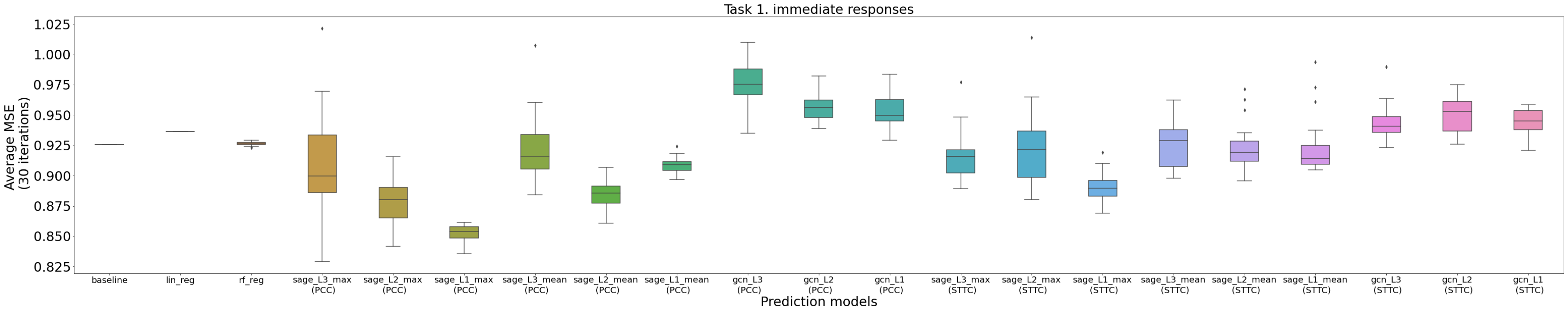

Figure S9. Average MSE (task 1. immediate responses) Box-plot representation of Table S5. task 1.

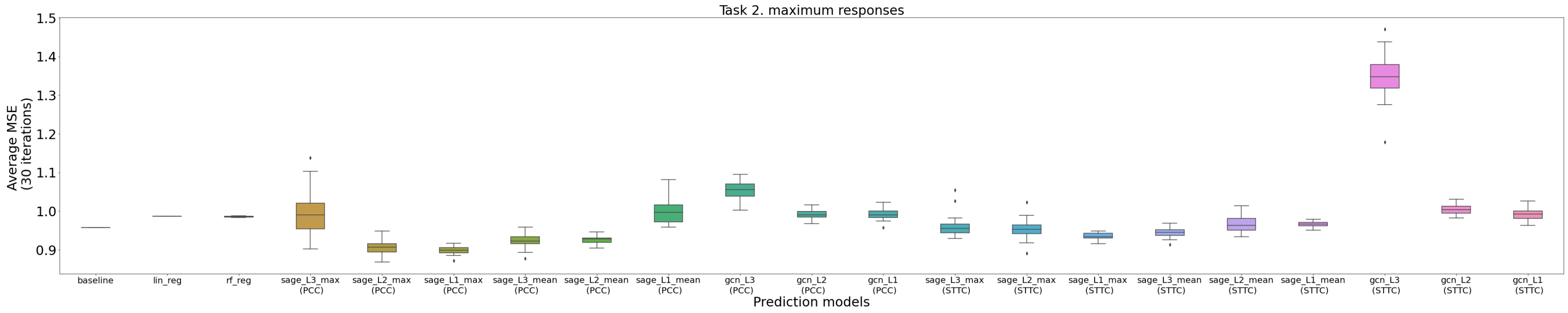

Figure S10. Average MSE (task 2. maximum responses) Box-plot representation of Table S5. task 2.

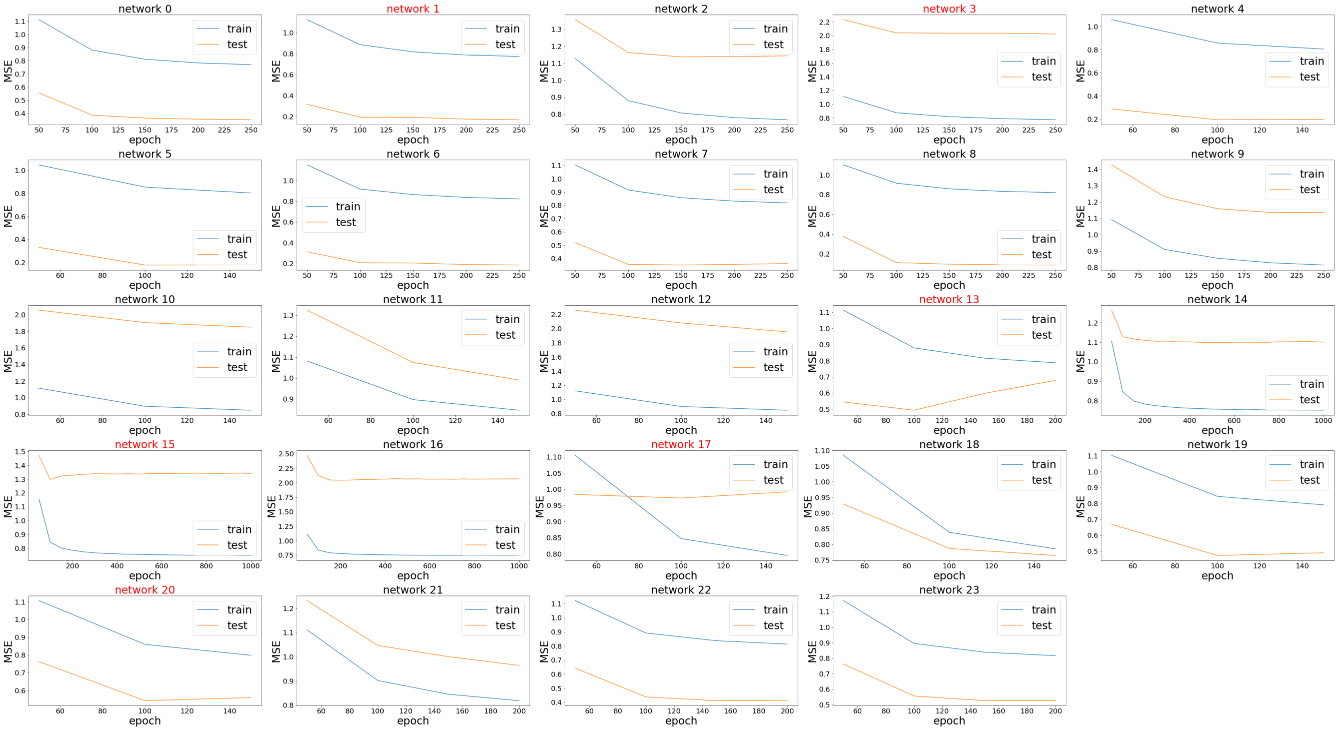

Figure S11. Loss curves for each network (task 1. immediate responses, GraphSAGE-1-conv, max pooling (PCC)) Each subplot shows loss curves measured during MSE (training, testing data) for each network. Subplots with red titles indicate networks that showed lower average MSEs than those of the baseline model.

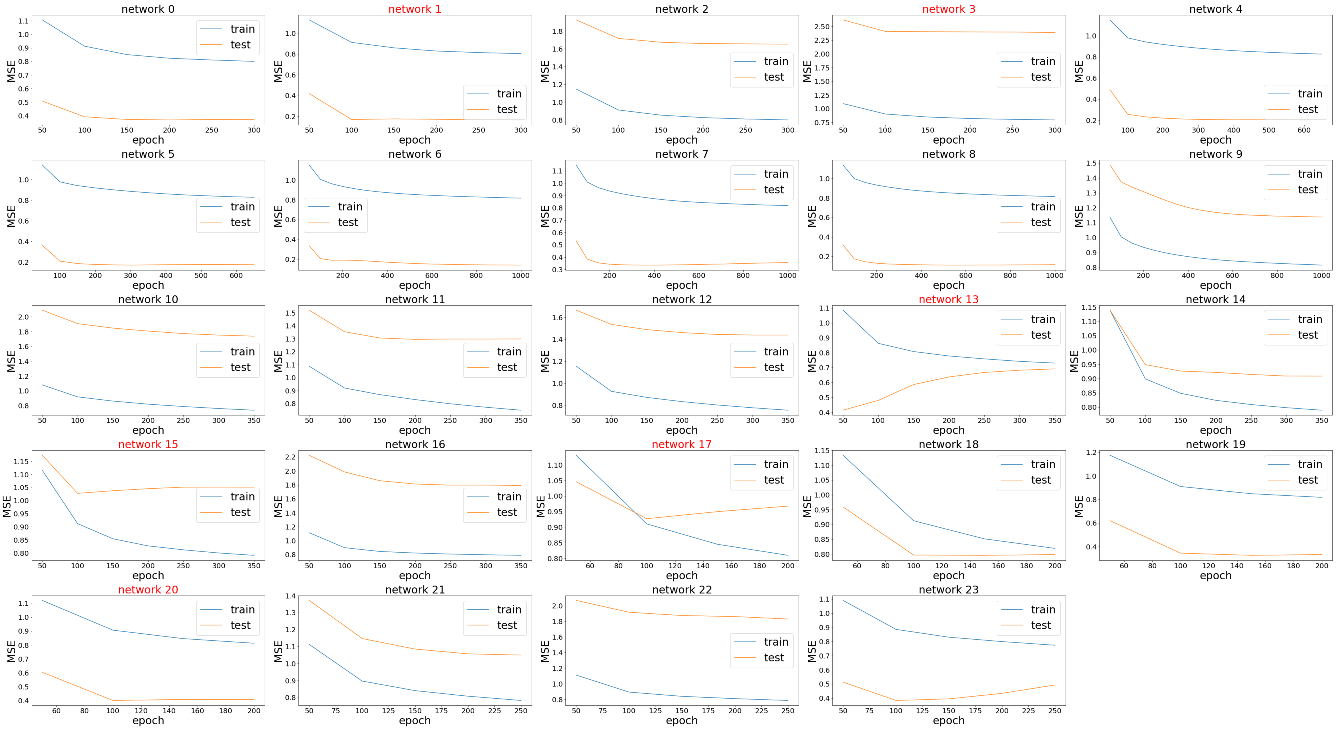

Figure S12. Loss curves for each network (task 2. maximum responses, GraphSAGE-1-conv, max pooling (PCC)) Each subplot shows loss curves measured during MSE (training, testing data) for each network. Subplots with red titles indicate networks that showed lower average MSEs than those of the baseline model.

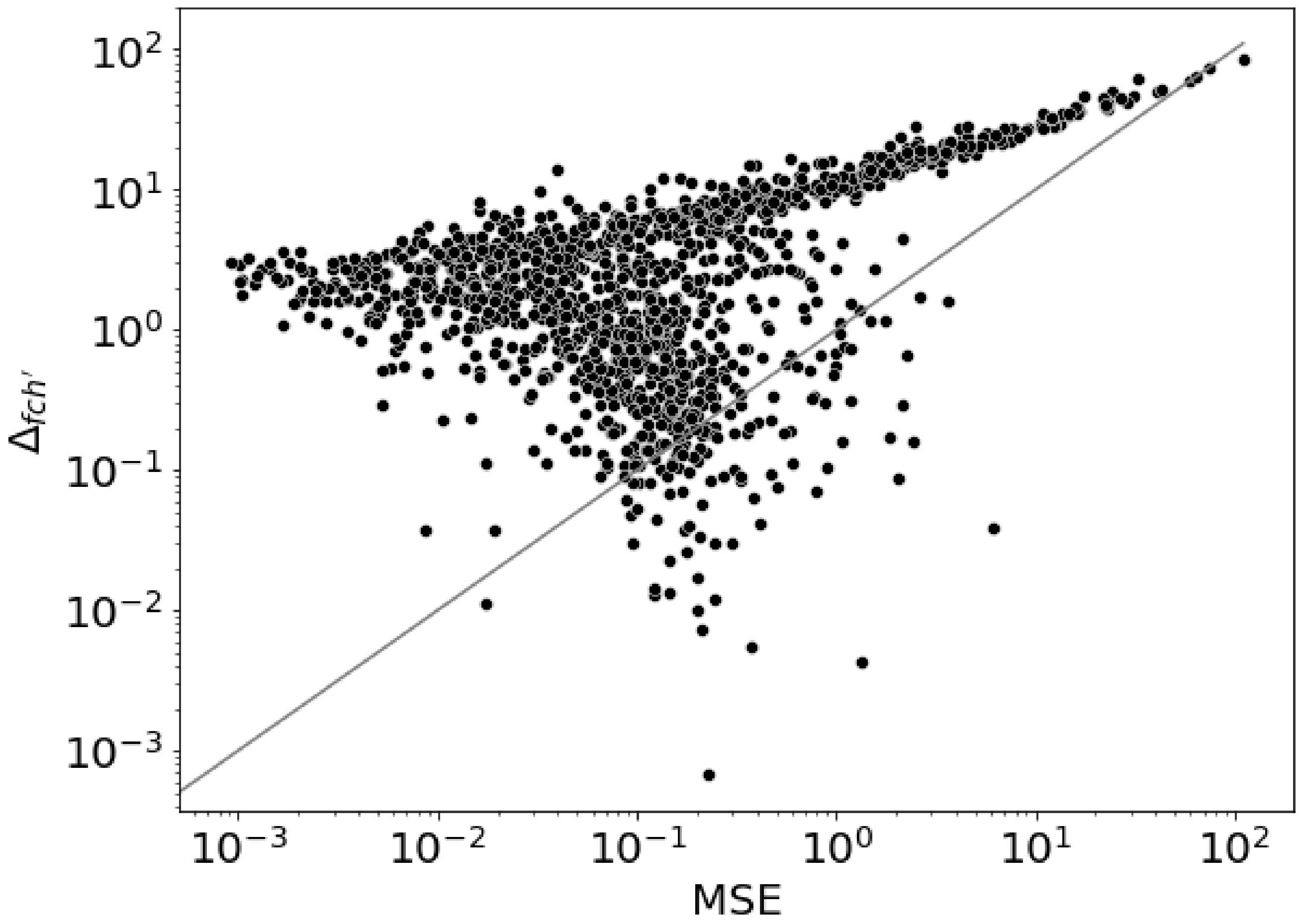

Figure S13. Target firing rate fold-change($\Delta_{fch'}$) vs MSEs (n=1695) Log-scaled. Neurons showing negative $\Delta_{fch'}$ are not depicted.

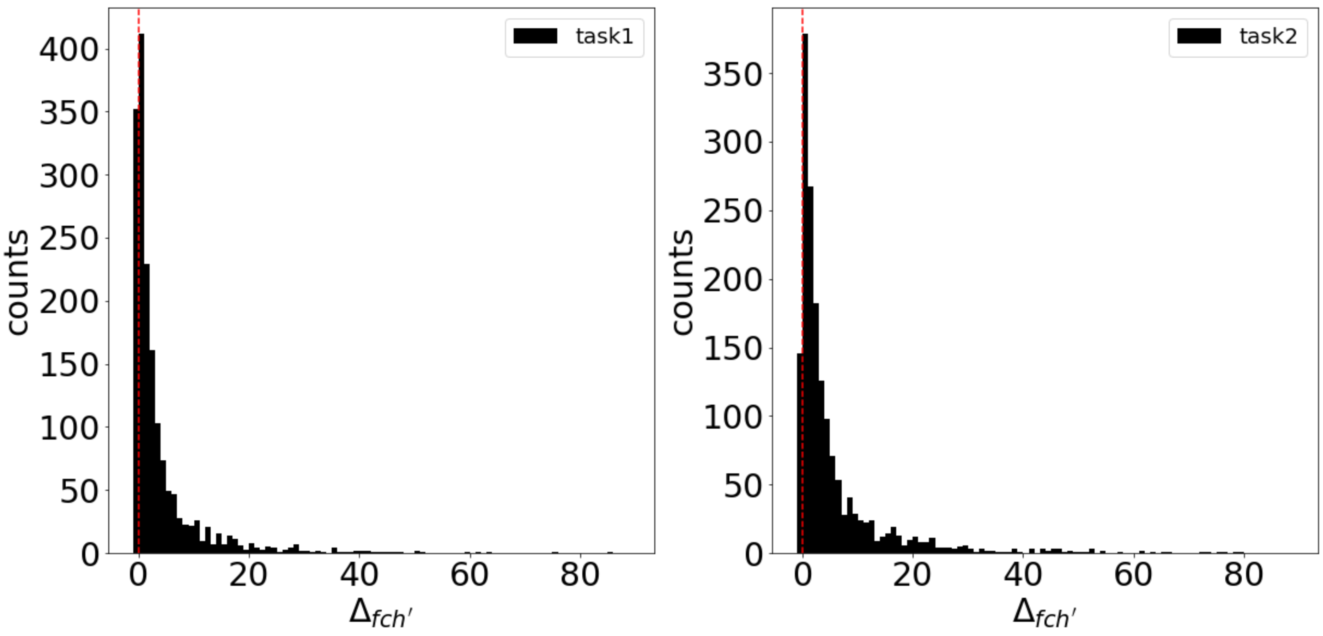

Figure S14. Skewed distribution of target variables

1 ASSESSING SPARSE FUNCTIONAL CONNECTIVITY

We implemented the maximum entropy model (MEM) using Minimum Probability Flow (MPF) (Sohl-Dickstein et al., 2011). While using default parameters stated in the "allbitflipextension" model [1], we tested 3 different bin sizes (5, 10, 20 ms) to generate binary spike trains. This bin size selection was motivated by the previous study by Das and Fiete (2020). The estimated functional connectivity (coupling matrix, J) was L2-normalized and used for the downstream analysis.

We tested the performance of MEM functional connectivity using the main dataset (task 1. immediate response). As shown in Table S13, none of the GraphSAGE implementations showed significant improvement over the baseline (two-sided paired sample t-test, $\alpha = 0.01$). We further tested different variants including unnormalized graphs and graphs without negative weights (by discarding all negative edges or subtracting the smallest edge weight from the graph). None of these variants resulted in improved accuracy (data not shown).

To probe the usefulness of MEM further, we generated a functional connectivity graph linearly interpolated between the Pearson correlation based graph and MEM based graph. Given that MEM with 5 ms showed the best accuracy with GraphSAGE-3-conv (max pooling) among other MEM instances, we selected functional connectivity derived from MEM with 5ms to mix it with Pearson correlation-derived functional connectivity graph. We computed the prediction accuracy of the best-performing model (GraphSAGE-1-conv, max pooling).

[1] URL: `https://github.com/Sohl-Dickstein/Minimum-Probability-Flow-Learning/blob/master/MPF_ising/K_dK_ising_allbitflipextension.m`

Table S13. MSE of Maximum entropy models with different bin sizes The table shows MSEs of GraphSAGE models using Maximum entropy model-derived functional connectivity. We generated binary spike trains using three different bin sizes(5, 10, 20ms)

**Models**	Task 1. Immediate response
Baseline	1.083
Linear regression	1.091
Random forest regression	1.076 $\pm$ 0.001
GraphSAGE-1-conv (max pooling, 5ms)	1.089 $\pm$ 0.007
GraphSAGE-2-conv (max pooling, 5ms)	1.098 $\pm$ 0.011
GraphSAGE-3-conv (max pooling, 5ms)	1.074 $\pm$ 0.011
GraphSAGE-1-conv (mean pooling, 5ms)	1.084 $\pm$ 0.006
GraphSAGE-2-conv (mean pooling, 5ms)	1.104 $\pm$ 0.01
GraphSAGE-3-conv (mean pooling, 5ms)	1.102 $\pm$ 0.014
GraphSAGE-1-conv (max pooling, 10ms)	1.166 $\pm$ 0.024
GraphSAGE-2-conv (max pooling, 10ms)	1.103 $\pm$ 0.016
GraphSAGE-3-conv (max pooling, 10ms)	1.126 $\pm$ 0.017
GraphSAGE-1-conv (mean pooling, 10ms)	1.085 $\pm$ 0.007
GraphSAGE-2-conv (mean pooling, 10ms)	1.096 $\pm$ 0.011
GraphSAGE-3-conv (mean pooling, 10ms)	1.121 $\pm$ 0.017
GraphSAGE-1-conv (max pooling, 20ms)	1.082 $\pm$ 0.003
GraphSAGE-2-conv (max pooling, 20ms)	1.099 $\pm$ 0.007
GraphSAGE-3-conv (max pooling, 20ms)	1.1 $\pm$ 0.011
GraphSAGE-1-conv (mean pooling, 20ms)	1.081 $\pm$ 0.004
GraphSAGE-2-conv (mean pooling, 20ms)	1.098 $\pm$ 0.025
GraphSAGE-3-conv (mean pooling, 20ms)	1.084 $\pm$ 0.008

In the following discussion, these acronyms and formula will be used:

PCC = Pearson correlation-derived functional connectivity

MEM = Maximum entropy model-derived functional connectivity

β = mixing strength

Weighted FC = β *PCC + (1-β) *MEM

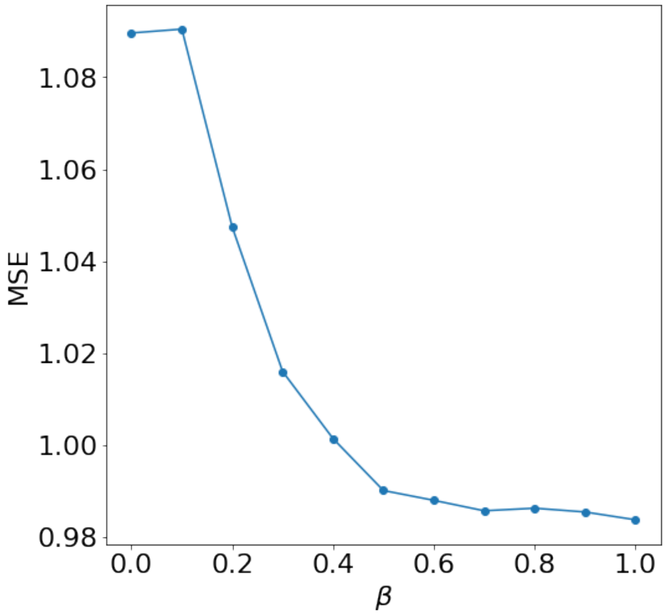

Figure S15. MSE of GraphSAGE-1-conv with max pooling, using linearly interpolated functional connectivity graphs. Each value of β yields a different graph.

With $\beta = 0$, we recover MEM. As shown in Figure S15, as we increase the proportion of PCC, we see an improvement in terms of MSE (decrease in MSE).

To understand why networks inferred by the MEM approach did not yield more information, we analyzed the edge weight distribution. Compared to PCC, MEM generated a lot of near-zero values. By clipping the edge weights under 0.01 (i.e. by applying an absolute threshold) for both MEM and PCC, we see a clear contrast in graph density between MEM and PCC for all 24 recordings (Figure S16 below).

We found that the MEM graphs were effectively sparse compared to the PCC graphs. This sparsity in the graph might limit the expressivity of GNN models as only few edges can pass the information in the MEM graphs.

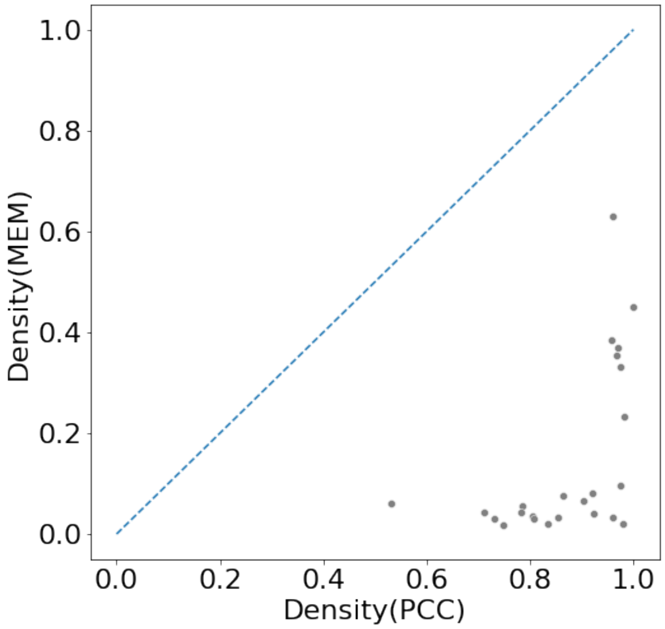

Figure S16. Comparison of clipped graph density between graphs derived using MEM and PCC. Each point represents a recording (or a network) and it is plotted in the figure using the density of the PCC-derived FC graph (as X-axis) and the density of the MEM-derived FC graph (as Y-axis). If both graphs had the same density, then the point would be plotted over the diagonal.

2 CONNECTEDNESS IN DIRECTED FUNCTIONAL CONNECTIVITY GRAPHS

We probed the connection density of directed FC without discarding smaller graph components in order to consider the same number of neurons as in undirected FC. Figure S17 shows the number of graph components and graph densities against the applied sensitivity γ for 24 recordings (or networks).

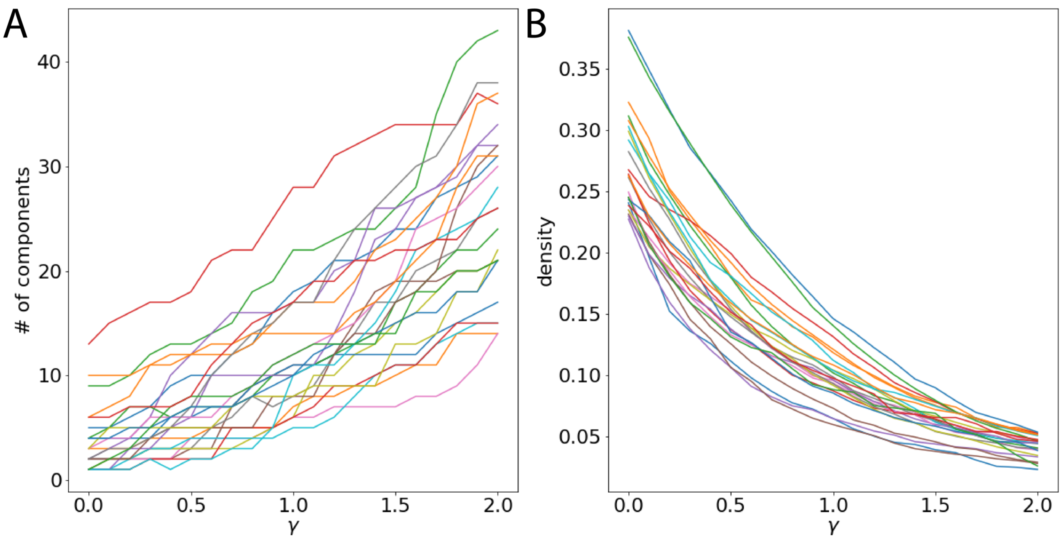

Figure S17. (A) The number of disconnected graph components against the sensitivity parameter γ. (B) Graph density against the sensitivity parameter γ.

As γ increases (higher threshold), we obtain graphs with lower connection density. We then compared the density of PCC-derived FC against directed FC thresholded with the lowest threshold value (γ = 0, using the population mean across all edge weights (before thresholding) as a threshold. Refer to Figure S18 below. Graph density for undirected graphs was computed as $D_{\text{und}} = \frac{2|E|}{|V|(|V|-1)}$, where $|E|$ is the number of edges and $|V|$ is the number of nodes in the graph. Graph density for directed graphs was computed as $D_{\text{dir}} = \frac{|E|}{|V|(|V|-1)}$. We additionally converted the directed FC graphs into undirected graphs and compared densities.

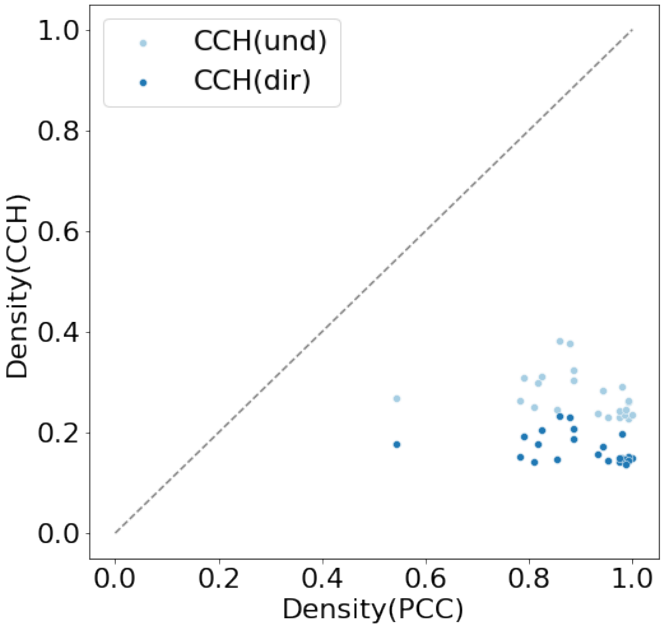

Figure S18. Comparison of graph density between directed functional connectivity (γ = 0) and PCC-derived FC. Each point represents a recording and it is plotted in the figure using the density of the PCC-derived FC graph (as X-axis) and the density of the CCH-derived FC graph with $\gamma = 0$ (as Y-axis). "CCH(und)" represents the density of directed FC graphs when converted into undirected graphs and "CCH(dir)" shows the true density of the directed FC graphs.

Figure S18 shows that the connection density of directed connectivity (CCH) was lower compared to the connection density of Pearson correlation (PCC) when comparing graphs of the same size (i.e., same number of neurons).

REFERENCES

Das, A. and Fiete, I. R. (2020). Systematic errors in connectivity inferred from activity in strongly recurrent networks. *Nat. Neurosci.* 23, 1286–1296

Sohl-Dickstein, J., Battaglino, P. B., and DeWeese, M. R. (2011). New method for parameter estimation in probabilistic models: Minimum probability flow. *Phys. Rev. Lett.* 107, 220601
